# Age-related effects on a hierarchical structure of canine cognition

**DOI:** 10.1007/s11357-024-01123-1

**Published:** 2024-03-21

**Authors:** Zsófia Bognár, Borbála Turcsán, Tamás Faragó, Dóra Szabó, Ivaylo Borislavov Iotchev, Enikő Kubinyi

**Affiliations:** 1 Department of Ethology, ELTE Eötvös Loránd University, Budapest, Hungary; 2 MTA-ELTE Lendület “Momentum” Companion Animal Research Group, Budapest, Hungary; 3 ELTE NAP Canine Brain Research Group, Budapest, Hungary

**Keywords:** Cognition, Aging, Cognitive structure, General cognitive factor, Personality, Health

## Abstract

The current study investigates whether there are statistically independent age-related influences on the canine cognitive structure and how individual factors moderate cognitive aging on both cross-sectional and longitudinal samples. A battery of seven tasks was administered to 129 pet dogs, on which exploratory and confirmatory factor analyses were employed to unveil the correlational structure underlying individual differences in cognitive performance. The best-fitting model featured a hierarchical structure with two first-order cognitive domains (individual problem solving, learning) and a second-order common factor. These higher order factors exhibited consistency over a period of at least 2.5 years. External validation linked the common factor positively to discrimination and reversal learning performance, exploration, neophilia, activity/excitability, and training level while negatively to cognitive dysfunction symptoms, suggesting that it is a good candidate for a general cognitive factor (*canine g*). Structural equation models identified three distinct age-related influences, operating on associative learning, on memory, and on *canine g*. Health status moderated the negative age-*canine g* relationship, with a stronger association observed in dogs with poorer health status, and no relationship for dogs in good health. On a longitudinal sample (*N* = 99), we showed that the direction and magnitude of change in *canine g* over up to 3 years is affected by various interactions between the dogs’ age, communication score, baseline performance, and time elapsed since the baseline measurement. These findings underscore the presence of a general cognitive factor in dogs and reveal intriguing parallels between human and canine aging, affirming the translational value of dogs in cognition and aging research.

**Supplementary Information:**

The online version contains supplementary material available at 10.1007/s11357-024-01123-1.

## Introduction

In recent years, several efforts have been undertaken to characterize the cognitive aging process in dogs, both behaviorally and physiologically [1–8]. The significance of these studies for veterinary science is evident, as older dogs, similar to humans, can suffer dementia-like symptoms [9, 10]. This similarity is also among the leading arguments for using the dog as a translational model in understanding human (cognitive) aging, especially since most model organisms do not naturally develop such syndromes. Beyond clinical analogies, dogs also exhibit a high phenotypic similarity to aged humans. Studies have revealed that aged dogs express a natural decline in various cognitive functions, including decreased attention, trainability, and memory, slower adaptation to new rules, and reduced responsiveness to known command words [5–8, 11–15]. However, there are two lines of research concerning cognitive ageing that have rarely been investigated in dogs so far (if ever). The first is the number of distinct age-related influences on different cognitive measures and the second pertains to how individual and environmental factors affect cognitive aging.

Cognitive aging in dogs is typically studied one task at a time, even in studies that observe performance across a broader range of tasks [8, 16, 17]. However, by investigating the effect of age separately for each task, the researchers assume not only that performance in different cognitive tasks is independent of each other but also that age-related effects on one task are independent of those on others, which may not necessarily be the case. In humans, cognitive performances in tests that tap into different cognitive domains have been found to be positively correlated, forming the so-called “positive manifold” [18]. It means that individuals that do well in one type of cognitive test tend to perform well in others as well. While there is still some disagreement regarding the causes of these positive correlations between tasks (see discussion by, e.g., [19, 20]), the current consensus leans toward a hierarchical structure model of cognitive abilities (e.g., [21, 22]). Raw task performances are organized into first-order factors corresponding to distinct cognitive domains, and on top of the hierarchy, there is a common underlying factor known as general mental ability, general cognitive factor, or simply the “*g*” factor, which accounts for the variance common to the first-order abilities.

Just as task performances are not independent from each other, neither are the age-related influences on these task performances. Although a large number of cognitive tasks have been found to be negatively related to age, and most age effects were shared across multiple tasks and acted on higher level domains, only a few of these tasks had a unique age-related influence. For example, [23] and [24] demonstrated across multiple independent samples and with different combinations of cognitive variables that only three statistically distinct negative age-related influences operated on the structure: on a first-order episodic memory factor, on a first-order perceptual speed factor, and finally, on the highest, second-order common factor (*g*). The existence of distinct age-related influences implies distinct (neurobiological) mechanisms in the background that need to be accounted for when attempting to explain age-related decline in cognition. Although there are speculations attempting to associate age-related influences with particular neural activity and neurobiological substrates [23], confirming such connections in humans poses challenges, as direct, long-term manipulation of neurobiological factors is not feasible in humans. Animal models can help tackle these links, and among them, the prime candidate is the dog. If higher level cognitive factors also exist in dogs, their common influence might be behind (some of) the age associations found in narrower abilities, making them suitable for such research endeavors. However, before investigating this, we must demonstrate that dogs also possess higher order cognitive domains that encompass the performance of multiple cognitive tasks.

While dogs rank among the most studied species regarding their cognitive capacities [25], these studies are typically comparative in nature, focusing on differences between specific dog groups (e.g., [11, 26]) or between dogs and other species [27, 28]. Even though most cognitive tests revealed vast individual differences in the dogs’ performance [29–31], researchers have rarely considered the possible causes and underlying mechanisms of this individual variation [32]. Consequently, little is known about the structure of cognitive abilities.

The first of the few attempts in this direction [33] found some indication of a positive manifold across response latencies in three cognitive tasks in beagles, but they did not carry out any further statistical analysis. Subsequent studies [34–37] generally concurred that instead of a single general cognitive factor, multiple domains of cognition are better suited to explain individual differences in performance. However, it is important to note that when describing individual variance in test performance, the existence of multiple cognitive domains alone does not exclude the possibility of a general cognitive factor above them. In the aforementioned dog studies, cognitive variables were subjected to a single exploratory factor analysis (EFA), and only first-order factors were extracted. The authors did not explore whether these extracted factors share any variance with each other, which could indicate the presence of a higher order latent factor. Furthermore, all studies used rotation when extracting the factors (mostly an orthogonal type), which could obscure the presence of a common underlying factor [38]. Moreover, none of these studies reported the reliability of the cognitive tasks, even though tasks with low within-individual reliability can obscure the cognitive structure and hinder the detection of a *g* factor [21, 39]. Finally, the majority of previous studies relied solely on EFA to extract performance factors, which is an inadequate tool for this purpose as it is not a reliable instrument for causal inference [40–42]. Confirmatory factor analysis (CFA) is better suited for testing whether the cognitive factors extracted are indeed causal determinants of the test performance [43]. CFA can partition the variance of the observations to test whether the hypothesized latent factors indeed account for the correlations among the observations.

CFA has not been used in dog studies to validate factors extracted from EFA except for one study. Arden and Adams [44] used this method to explore various alternative models of cognitive structure and found some evidence for a hierarchical structure with a human-analogue *g* factor at the apex. However, their battery consisted of only three tasks, which did not allow for testing the structure of a broad variety of cognitive abilities [45]. One of their tasks had low reliability, suggesting that a higher portion of individual variance could be attributed to random or measurement error [39], potentially weakening the correlation matrix [21]. Moreover, their sample consisted only of border collies (with a relatively low sample size), limiting how much inter-individual variability could be captured.

Before investigating whether there are statistically distinct age-related influences operating at different levels of cognitive hierarchy, it is imperative to address these methodological shortcomings of previous studies and analyze the correlational structure underlying individual differences in dog cognition to extract higher order cognitive factors that encompass multiple specific task performances. This was the first aim of the current study.

The second aim of our study was to investigate how the individual and environmental characteristics of the dogs influenced the effects of age on cognitive performance. Similar to humans, there are large individual differences in the dogs’ aging curves, which may partly be attributed to successful (healthy) and unsuccessful (abnormal) aging processes (e.g., [13, 46–48]). Previous studies rarely investigated which factors affect the cognitive aging processes in dogs, despite extensive research on this topic in humans. For instance, physical and mental activity, education, and (premorbid) IQ consistently emerge as significant predictors for the onset of human dementia, while sex, age, ethnicity, and geographic region are identified as moderating factors [49–53]. Given all the parallels between human and dog aging, combined with the overlap in their natural environments and factors shaping their aging processes [54], one might expect that similar individual and environmental factors would also influence the aging curves of dog cognition as human cognition. There are two primary approaches to investigate this.

The most common method to test age-related changes is the cross-sectional design, which offers several advantages over the longitudinal approach, including a higher sample size, a broader age range in the sample, and faster data collection. However, the results may be influenced by a cohort effect, and this method can only elucidate aging dynamics at the population level, not at the individual level. To study individual differences in the aging curve of cognition, longitudinal research is required [55]. Such studies can also identify factors influencing the differences in the onset and trajectory of age-related decline and disentangle the effects of age and inherent differences in cognition on the overall performance. In our study, we employed both methods. Using a cross-sectional approach, we investigated the age trajectory of the extracted higher order cognitive factor(s) and explored the modifying effects of owner-reported individual features (such as personality, keeping conditions, activity and training routines, and owner attitude) on this association. In a longitudinal sample, we analyzed the long-term reliability (temporal consistency) of the extracted higher order cognitive factor(s) across time, as well as individual differences in the change in cognitive performance and the factors that affect the magnitude and direction of this change over time.

## Methods

### Ethics statement

The procedures applied complied with national and EU legislation and institutional guidelines, and the study was performed under the recommendations of the International Society for Applied Ethology guidelines for the use of animals in research. A written statement (PE/EA/853–2/2016) was obtained from the local ethical committee (“Pest Megyei Kormányhivatal Élelmiszerlánc-Biztonsági és Állategészségügyi Igazgatósága”, Budapest, Hungary). According to this statement, the current study is a non-invasive observational experiment and thus is allowed to be performed without any special permission according to the corresponding definition by law (‘1998. évi XXVIII. Törvény’ 3. §/9. — the Animal Protection Act). The owners of dogs who volunteered to participate in the study were informed about the procedures and data handling protocols, provided written consent for their dogs’ participation, and could at any point decline to participate.

### Subjects

#### Cross-sectional sample

*N* = 129 dogs participated in the cognitive test battery. The dogs’ ages ranged from 2.61 to 14.54 years (mean age ± SD = 8.38 ± 3.21 years), 48.8% were males, and 77.5% were neutered. The sample consisted of 59 mixed-breed dogs and 70 purebred dogs from 33 different breeds. All dogs were middle-sized, their weight ranged from 7 to 45 kg, except for one dog weighing 80 kg (mean weight ± SD = 21.68 ± 7.86 kg). Detailed raw data can be found in SI 1. Before participating in the test battery, the dogs underwent assessments to ensure they were eligible for participation. Their motor skills were evaluated by a qualified physiotherapist, and a sensory assessment was conducted to exclude subjects with potential visual and/or acoustic impairments, following recommendations by [56]. Only animals without major sensory impairments or conditions that hindered mobility or prevented them from completing the tasks or detecting stimuli were included in the study. Additionally, the dogs were required to be free from overt signs of neurological and other physical health problems, as reported by the owners.

A subset of dogs (*N* = 59, mean age ± SD = 8.71 ± 3.47 years, 42.4% male) participated in an independent cognitive measure involving discrimination and reversal learning, on average 13 days (ranging from 1 to 94 days) after completing the cognitive test battery. These tasks assess associative learning, behavioral flexibility, and inhibition, key components of general intelligence in humans and other animals (as reviewed in [45, 57]). This dataset was utilized to validate the extracted cognitive domains.

#### Longitudinal sample

A subset of *N* = 99 dogs participated in the test battery multiple times, with an average interval of 1.26 ± 0.87 years (range: 0.23–2.95 years) between the test sessions. Among them, *N* = 41 dogs participated twice, *N* = 32 dogs participated three times, *N* = 19 dogs participated four times, and *N* = 7 dogs participated five times. The procedure and the tasks’ rank order were the same in all test sessions. Dogs’ age at first participation ranged from 2.61 years to 14.54 years (mean age ± SD = 8.83 ± 3.02 years), 49 of them were males (12 intact) and 50 were females (three intact). The sample consisted of 48 mixed-breed dogs and 51 purebreds, with weights ranging from 7 to 44 kg (mean weight ± SD = 20.39 ± 8.56 kg), except for one dog weighing 80 kg. Among these dogs, *N* = 70 also took part in a 3-month-long intervention therapy between the test sessions (*N* = 10 were only in the control group). We included these measurements in the subsequent analyses due to the negligible effect of the intervention therapy on the dogs’ cognitive test performance [58]. Parts of this longitudinal sample were utilized in the following analyses:

For the task reliability (repeatability) and confirmatory factor analysis, we utilized the second test sessions with a between-session interval no longer than 0.5 years (*N* = 72 dogs, mean age ± SD = 8.93 ± 3.00 years at their first test session, with 47.5% male). We used all longitudinal data to estimate the reliability of cognitive measures over time and also to conduct a longitudinal assessment of changes in the cognitive factors. However, due to errors during the test, either in the video recording or by the experimenter, we could not calculate the higher level factor scores for three measurements: specifically, the second session for two dogs and the fourth session for one dog. Consequently, we excluded these measurements and conducted the analyses on *N* = 187 measurements from *N* = 99 dogs.

### Behavior tests

The cognitive test battery comprised ten tasks in total and occurred in two experimental rooms: room 1, measuring 5 × 6 m, and room 2, measuring 3 × 5 m. The tasks followed a fixed order for all subjects to standardize any carry-over effects between them. The entire battery typically lasted around 60 minu, including a short break (Movie S1).

Seven tasks were designed to assess distinct cognitive abilities. These tasks were adapted or modified from published test batteries, required no pre-training and aimed to be repeatable with minimal habituation or learning effects. These seven tasks were used to analyze the correlational structure underlying individual differences in dog cognition and extract higher order cognitive factors. The remaining three tasks served to validate these factors externally. The first two tasks of the battery evaluated the dogs’ activity and exploratory tendencies, while one task (novel object recognition) aimed to assess the dogs’ neophilia.

#### Cognitive tasks

1. *Pointing* [59]

##### Aim:

To assess the dogs’ (hereafter referred to as D) ability to follow a momentary human pointing gesture when locating a hidden food reward, and also D’s ability to shift from a previously rewarded response by pointing to the same side three times in a row, then to the other side during the subsequent three trials.

##### Procedure:

The task was conducted in room 2. In the first phase (warm-up trial), we aimed to ensure that D was comfortable approaching and eating from the containers (pots) used in the test trials. Upon entering the room, the owner (hereafter referred to as O) sat down on the chair at the starting position (3 m from the position of the experimenter (hereafter referred to as E), took off the leash and held D by the collar. E called D’s attention by saying, “D name + look,” showed the treat, then dropped it into the pot and put it in front of her on the ground. Then, D was released and allowed to take the food. O and E were allowed to encourage D if necessary. In the second phase (test trials), E carried out six test trials. Each started with O sitting in his/her chair, holding D by the collar at the starting position, and E standing 3 m from them, holding two identical pots folded into each other. E called D’s attention, and after establishing eye contact, she showed the treat and dropped it into the upper pot. She then shuffled the two pots 2–3 times so that D could not know which one contained the food. Next, she placed them on the marks on the floor to her left and right (the distance between the two pots was 1.5 m). She then called D’s attention again (“D name + look”) and performed a momentary distal pointing gesture (3 s) to the baited pot. After E returned to her starting position (both hands held in front of her chest), O released D. After D made a choice (its nose came within 10 cm of the pot), E removed the other pot before D had the chance to investigate it. If D made a correct choice, it was allowed to eat the food. If D made an incorrect choice, the baited pot was only shown to D. If D did not make a choice due to an external disturbance or O error, E repeated the given trial. If the lack of choice was not caused by any disturbance, it was counted as an incorrect trial, and the test continued. At the end of the trial, O called or led D back to the start position, and the next trial began. There were six test trials, with the first three trials on the same side and the second three on the other side. The location of the first baited pot was counterbalanced among dogs.

2. *Manipulative persistency* [60]

##### Aim:

To measure D’s willingness and ability to obtain treats from an interactive toy and its persistence in trying to obtain an inaccessible food reward.

##### Procedure:

The task was conducted in room 2. In the first phase (solvable trial), E showed the toy to D (Kong Wobbler™, small or large, depending on D’s size), then baited the toy in front of D with 20 pieces of small-sized treats, which could be retrieved by manipulating the toy. The toy was then placed in the middle of the room, and D had 60 s to manipulate it while E and O remained in the same positions as in the previous test. If D lost interest, O was allowed to encourage it verbally and by pointing at the toy without leaving his/her chair. In the second phase (unsolvable trial), the test was repeated with the same procedure, but this time, E baited the toy with a large treat that D could not obtain. After this test, there was a break (5–10 min) during which D remained outside of the test rooms. Then, room 1 was rearranged, removing all the objects.

3. *Clicker game* [61]

##### Aim:

To measure D’s associative learning ability and behavioral flexibility, that is, its ability and willingness to offer novel behaviors to E in a positive reinforcement setup.

##### Procedure:

The task was conducted in room 1. O sat on the chair next to the door and was asked not to communicate or interact with D during the test. E stood in the center of the room with a food pouch filled with sausages on her belt and holding a sound-making device (similar to a clicker but displaying a different sound). She called D to her, then asked it to sit. Once D sat in front of her, she clicked and threw a piece of sausage on the floor. After that, E remained motionless but clicked and rewarded D for presenting any object- and body-related novel behavior. If D presented the same behavior repeatedly, E waited until a new behavior was offered while also encouraging D by smiling and nodding but without speaking. The test lasted for 2 min, measured from the first click sound.

4. *Problem-solving* [62]

##### Aim:

To assess D’s individual problem-solving when locating a hidden food reward in a problem box, together with D’s behavioral inhibition and flexibility, by systematically shifting the visibility of the food reward and the location of the opening on a problem box.

##### Setup:

The task was conducted in room 1. The apparatus had the following dimensions: a 62.5 cm × 53 cm platform with a 22.5 cm × 22.5 cm × 38 cm rectangle box (opaque or transparent) attached to it. The box was closed on the top, bottom, and three sides, with only one side left open.

##### Procedure:

In all trials, O sat on a chair 1.5 m from the apparatus, held D by the collar until E baited the apparatus and returned to her starting position next to O. Then, O let D free, and D had 45 s to obtain the reward. A choice was defined when D’s head touched the box, and a successful trial was defined when D’s head was inside the box. O was allowed to encourage D verbally and by pointing at the apparatus without leaving his/her chair. If D failed in a given trial, E provided the minimal necessary help to D to get the reward to prevent loss of motivation.

In the first phase (ppaque, trials 1–3), the apparatus was opaque (wood), D could see the baiting process, and the opening was always in the middle position, facing away from D.

In the second phase (transparent, trials 4–10), the apparatus was transparent (plexiglass) (so D was able to see the food reward inside). In these trials, E prevented D from seeing the baiting process (i.e., the location of the opening on the apparatus) via a visual barrier. The location of the opening was on the same side (left or right) in trials 4 to 6, shifted to the opposite side (right or left) in trials 7 to 9, and then shifted to the middle position (facing away from D) in trial 10. The location of the opening in trial 4 was counterbalanced among subjects.

5. *Attention* [1, 7]

##### Aim:

To measure D’s attentional capture and sustained attention in social and non-social contexts.

##### Setup:

The task was conducted in room 1. O sat on a chair approximately 4.5 m from the wall where the stimuli were presented. O was told to ignore both D and the actions of E. D was positioned next to O at the beginning of each context and remained leashed during the entire test.

##### Procedure:

The order of the two trials was counterbalanced across subjects. In the non-social trial (flying object), E remotely manipulated a yellow plastic frisbee from outside the room by pulling a fishing line through a metal loop in the ceiling in the testing room. The object moved up and down (seemingly on its own) next to the wall facing D for 1 min. In the social trial (“painting” the wall), E entered the testing room, silently walked to the wall, and, with her back to D, made up-down movements (as if painting the wall). After 1 min, E left the room without looking at D.

6. *Training for eye contact* [1, 11, 61]

##### Aim:

To measure D’s associative learning ability to sustain social attention, that is, to learn the association between establishing eye contact with E and food reward, and then sustain eye contact with E for increasing durations.

##### Procedure:

The task was conducted in room 1. In the first phase (training), D was unleashed, O sat on a chair next to the door and was instructed to ignore D. E stood in the center of the room holding a clicker-like device in one hand, with both hands positioned in a relaxed posture by her sides. E had a food pouch on her belt, positioned at her back. First, E called D’s attention and threw a piece of food on the floor. Then she remained motionless, and whenever D established eye contact with her, E clicked and tossed a piece of food on the floor. This phase lasted for 20 eye contacts or a maximum of 5 min. There were two conditions in the second phase (sustained eye contact): silence and with distraction, and the order was counterbalanced among dogs. In both conditions, D had to maintain eye contact with E for gradually increasing durations to receive a reward. Each condition had five levels: 2 s, 5 s, 10 s, 20 s, 40 s. Once D successfully passed a level, the latency between establishing eye contact with E and the click + reward was increased to the next level. D had three attempts to pass each level. If D failed all three attempts, the test was terminated. In the “with distraction” condition, white noise was played in the background (the mean sound pressure level of the playback was 49 dB). D received three eye contact retraining trials between the two conditions to maintain motivation.

7. *Memory* [5]

##### Aim:

To measure D’s visuo-spatial memory.

##### Setup:

The task was conducted in room 1. There were five identical pots on the floor, positioned at an equal distance (3 m) from D’s starting position, each pot 1.6 m from the other in a semi-circular arrangement.

##### Procedure:

E, O, and D entered the room, O and D walked to the starting position. E called D’s attention, showed a piece of food, walked to a preselected pot in a straight line from the start, and placed the reward in the pot. Then, E, O, and D left the room, and outside, O distracted D by giving simple commands or petting and talking to D. After 30 s, E, O, and D re-entered the room, returned to the starting position, and O released D. The trial ended when D found the treat. If D did not succeed in a given trial, E provided the minimal necessary help to D to get the reward to prevent loss of motivation. There were five trials in total, and each container was baited once in a predefined order. The order of the baited locations was counterbalanced across subjects.

#### Tests used for validating the extracted factors

We also aimed to externally validate the extracted cognitive factors by analyzing their correlation with performance in tests that should theoretically be related to the general cognitive factor. The dogs also participated in three additional tests assessing two known covariates of *g*: exploration tendencies and neophilia. High explorative behavior and low neophobia have been consistently found to be positively related to cognitive performance in different tasks across a wide range of species (as reviewed in [63–66]), including dogs [67]. A positive correlation between the common cognitive factor extracted by the statistical tests and exploration and neophilia would provide external validation for the common factor as a candidate for a general cognitive factor (*canine g*).

#### Exploration and box rustle [68]

These two tasks aimed to measure D’s activity and exploration in a room containing a wide range of objects. Both were conducted prior to the cognitive tasks, ensuring that individuals were unfamiliar with the test room, and their exploration pattern was not impacted by intervening experiences in other tasks of the battery.

#### Setup

Both tasks were conducted in room 1, which included 16 objects to explore. A chair was placed next to the wall, and four larger objects placed in the corners (a large cardboard box with a pink plastic bowl filled with plastic bags placed on top; a small table with a basket filled with plastic bags placed on top; a bedsheet with a small cardboard box on top filled with shredded paper; a waste bin filled with shredded paper) were present in all setups. The other 11 objects were selected from a pool of small-sized everyday objects with different colors and materials. These were placed in a circle around the middle of the room and along the walls.

#### Exploration test

O and D, on a leash, entered the room together. In the first (leashed) phase of the test, O stood for 20 s near the closed door without interacting with the leashed D. In the second (unleashed) phase, O took off the leash on E’s signal from outside and released D, ignoring D afterwards. For 120 s, D was free to explore the room while O remained standing next to the closed door. If necessary, O could give a release command to D at the beginning of this test.

#### Box rustle test

O moved slowly around the room, searching for four metal coins, one hidden in each of the four containers placed in the corners of the room. O was instructed to visit the locations in a fixed order (from right to left), spend at least 10 s at each location, and ignore D. D was free to move around. The total duration of this task was 60 s, and E’s signal from outside marked the end.

After this test, E entered the room and greeted the dog-owner pair. She also petted and played with D to ensure that D was not afraid of her and would be familiar with her presence in the subsequent cognitive tasks.

#### Novel object recognition [69]

This task was carried out at the end of the test battery, just before the memory task. It aimed to assess D’s reaction towards and potential preference for novel and familiar toys.

##### Setup:

The task was conducted in room 2. The toys used in the test were selected from a pool of six dog toys, and their combination was counterbalanced between dogs.

##### Procedure:

In the first phase (passive familiarization), E, O, and D entered the room, O sat down on a chair, let D free, and afterwards ignored D. There were two identical dog toys on the floor, 2 m apart from each other and 1.2 m from D. D was free to interact with them for 30 s. In the second phase (active familiarization), E approached and interacted with both toys (picked them up individually and engaged D’s attention by saying, “what do I have?” and “look at this!” in a happy voice). This phase also lasted for 30 s. After phase 2, O and D left the room for 5 min while E switched one of the toys to a new one. In the third phase (test phase), after re-entering the room, O sat down and released D, then D was free to interact with the toys for 60 s.

#### Discrimination and reversal learning [6, 70]

A subset of *N* = 59 dogs also participated in a discrimination and reversal learning task approximately 2 weeks after the cognitive battery. A positive correlation between the common cognitive factor and the dogs’ learning performances would provide evidence that the common factor represents a domain–general cognitive factor. The task was based on the cognitive bias paradigm and contained a discrimination phase, aiming to assess D’s ability to learn the association between the location of a stimulus and the reward, and a reversal phase, which measured D’s ability to re-learn the association when the positive and negative stimuli were reversed.

##### Setup:

The task took place in room 2. The stimulus was a blue plastic plate (20 cm in diameter), and the discrimination was based on the location of this plate (left- or the right-hand side of E). The side used as the positive stimulus (S +) was counterbalanced among the dogs, and when the bowl was placed on this side, it always contained a small piece of food, while on the negative side (S −), the bowl was always empty. D received the positive (S +) and the negative (S −) stimuli in consecutive trials presented in a fixed semi-random order (S + S + S − S + S − S −), which was repeated until the criteria (see below) was reached or for a maximum of 50 trials.

##### Procedure:

At the beginning of each trial, O was sitting on a chair approximately 3 m from E and held D by the collar or leash. E turned its back to O and D and baited (or pretended to bait) the plate. Then, she turned back and called D’s attention (“name + look’). Once she established eye contact with D, she put the plate on its predetermined location (left or right side, ~ 1 m from E). O was instructed to let D go immediately as the plate touched the floor. If D did not start moving when released, O was allowed to encourage it verbally (e.g., “Go!,” “It’s yours”) or by gently touching it. Apart from this, no other forms of communication were allowed. D had 15 s to reach the plate (and, in the case of S + , eat the food), then E picked up the plate, and O called D back for the subsequent trial.

In each trial, E recorded the latency to reach the plate, measured from the moment the plate touched the floor until D was < 15 cm of the plate (defined by markings on the floor). If D did not approach the plate, E gave the maximum latency (15 s). D was deemed to have learned the association between the stimulus and the food when the longest latency in the last five positive (S +) trials was shorter than any latency in the last five negative (S −) trials. The testing was terminated if D did not reach this criterion within 50 trials or refused to leave the chair’s proximity for three consecutive trials. If D passed the criteria of the discrimination phase, the test continued with the reversal phase after a short break. In the reversal phase, the procedure was the same, but the S + and S − locations were switched, i.e., if the S + was on the left side, it became the right side. Again, D had a maximum of 50 trials to learn the reversed association. The criterion of learning was the same as in the discrimination phase.

#### Test variables

The behavior tests were video-recorded, and the videos were later coded using the Solomon Coder program (beta 19.08.02) and a scoring sheet. In contrast to most human and animal general cognitive ability tests, we did not select a single reference variable a priori. Instead, we collected multiple variables for each task (Table S1 in SI2). We used a top-down approach and coded/scored a broad list of behavioral measures based on what was coded in the previous studies related to these tasks, as well as our preliminary observations (pilot tests). From this list, we excluded variables with low variability and/or with high skewness (caused by a few outlier values). Such variables are either not suited to detect individual differences in performance or not sensitive enough, thus differentiating only between the extremes. The criteria for exclusion were determined for each variable type separately. For durations (all with the hypothetical range of 0% to 100%), a variable was rejected if its range was < 50, and/or its mean was < 10. For latencies and frequencies, we excluded variables in which more than 50% of the dogs received the same value (mostly 0 or maximum). For nominal scores (all with four hypothetical discrete values: 0 to 3), a variable was excluded when more than 70% of the dogs received the same value or when a variable had a bimodal distribution (i.e., less than 10% of the dogs received the two least frequent values combined).

To assess the inter-observer reliability, we randomly selected a sample of 20 dogs in each task to be coded by a second observer. The inter-observer reliability was calculated using a intraclass correlation coefficient (ICC) for all variables, and we retained only those variables that have at least moderate reliability (ICC > 0.5) (Table S1 in SI2).

### Questionnaire

To collect basic information regarding the demographic attributes of the dog and the owner, as well as the social attributes of their interactions, we used a demographic questionnaire described in [14, 71]. All questionnaire items can be found in Table S2 in SI2. To measure dog personality traits, we used the five factors of the Dog Personality Questionnaire (DPQ [72]). We used 5-point Likert scales instead of a 7-point in the original 45 items, but this simplification did not lower the reliability, based on Wallis et al. (2020) report [14]. The factors were calculated by averaging the items (reversed if necessary) following the original structure published in [72].

Finally, the questionnaire included three additional queries: 10 questions about the owners’ attitude towards dogs, 30 items about the cognition and communication of the dog, originating mainly from [10, 73], and 11 items asking about the dogs’ character. The owners indicated their agreement/disagreement with each statement using a 4- or 5-point Likert scale in all these questions. The questionnaires were filled out around the date of the first test session (mean ± SD = 0.998 ± 3.61 months; range: − 14.95 to 15.15 months).

### Statistical analyses

#### Performance in the cognitive tasks and task reliability

First, to obtain composite scores reflecting the animal's test aggregate task performance in the cognitive tasks, we ran a principal component analysis (PCA) with oblimin rotation for each task separately, except for the pointing task, where only a single variable was coded. We decided on this method because, in contrast to humans and mice, no standardized protocols have yet been established to assess particular cognitive abilities. Thus, our battery may include tasks where the performance is strongly influenced by non-cognitive factors (i.e., motivation or training experience), tasks where the performance is related to multiple cognitive abilities, or where different abilities are engaged across different subjects. These task impurities can obscure the underlying structure in the individual variance [45]. Running a PCA for each task was a suitable way to filter out variables whose individual variance had different sources than the other variables. Moreover, extracting components that represent the common variance shared across multiple variables could also help reduce measurement error and other variable-specific effects [23]. In cases where the variables were categorical (attention task), the PCA was run based on a polychoric correlation matrix; otherwise, normal correlation matrices were used. The number of components extracted was decided by parallel analysis in all analyses. Variables that failed to load > 0.5 on any components were removed. We used Kaiser–Meyer–Olkin (KMO) measure and Bartlett’s sphericity test to determine the sampling adequacy and Cronbach’s alpha coefficient to assess the internal consistency of the items.

We extracted seven components in total (Table S1 in SI2). The KMO value was > 0.5 for all analyses. In 5 out of 6 task-level analyses, all variables loaded on a single component, suggesting that they all share a common source of variance. In one task (clicker game), we found two components.

The behaviors coded during the three tasks used for external validation (exploration, box rustle, and novel object recognition) were also subjected to PCAs to extract an aggregate measure of test performance (Table S1 in SI2). In the case of the novel object recognition test, two components emerged, and only the first indicated the dogs’ preference for the novel object; we expected a positive association with the common factor in the case of this component.

The task reliability was investigated using intraclass correlation (ICC, two-way mixed model). The pointing and the attention to object tasks were found to have low repeatability (Table S1 in SI2), which could mean that a higher portion of the individual variance in this task is due to random or measurement error or factors unrelated to cognition [39]. Thus, this task was excluded from further analyses. The reliability of the other five tasks (six components) was significant and at least moderate (ICC > 0.5), suggesting that the intra-individual variability was not too high for the majority of the tasks.

#### Correlation structure among the cognitive tasks

To explore and describe the correlation structure among the different tasks, we subjected the six components to exploratory factor analysis (EFA) using the principal axis factoring method of SPSS (version 28). We conducted the EFA both without rotation and with oblimin rotation. The former analysis aimed to identify the potential psychometric *g* based on the first unrotated factor (see [38, 74]). The rotated analysis aimed to assess if there are distinct cognitive domains, that is, specific groups of cognitive tasks which share a part of their variance with each other but not with other tasks. In this latter analysis, we removed the cognitive components that failed to load > 0.3 on any factors [75].

Since a part of the positive correlations among different cognitive measures could be due to the relationship of these measures to the same non-cognitive factor [23], and in the case of cognition, a primary candidate is age, we also investigated if the age of the dogs has a significant contribution to the positive correlations among the performance of various cognitive tasks. For this, we replicated the EFAs on the age residuals of the data. The age association of all six components was investigated using linear and quadratic regression models to obtain the age residuals. Since the age relations were primarily linear for all components (Table S3 in SI2), the residuals were extracted from the linear regression models, and we replicated all analyses on the age residuals of the data.

While EFA is well-suited to describe and summarize the correlation patterns among the variables, it is not well-suited to explain where the correlations among the observations originate from. Therefore, we also ran a confirmatory factor analysis (CFA) on the data using the AMOS program (version 27). Since it is statistically inappropriate to conduct both EFA and CFA on the same dataset [76], the CFA analyses were run only on the second test session data of *N* = 72 subjects. Similar to the EFA, the CFAs were also conducted both on the raw data and the age residuals of the data to assess the effect of age on the cognitive structure. Based on the results of the EFA, we fitted alternative models on the data selected from those described in [44]. The cognitive components were entered in all models as the observed variables (indicators), each with its unique error variance. We posited various numbers of higher order latent factors influencing the components according to the structure of the actual model. The residual covariances (i.e., covariances of the error variances) among the cognitive components, even those between components of the same latent factor, were fixed to zero, which posited that all correlations among the different tasks would be accounted for by their common latent factors. The models were compared using AIC and BIC values and two maximum likelihood criteria: Chi^2^ test, which evaluates the discrepancy between the model and the data, and root mean square error of approximation (RMSEA), which assesses the discrepancy between the predicted and observed values, so values closer to 0 correspond to better fit. We considered RMSEA < 0.1 as acceptable and < 0.05 as a good model fit [77]. Posterior predictive *p*-value (PPP) was used to reject models, where PPP < 0.05 indicates poor model fit. The best-fitting model structure was used in all subsequent analyses.

#### External validation of the extracted factors

We used structural equation models conducted with AMOS to determine if the individual’s activity, exploratory tendencies, and preference for novelty would correlate with the extracted cognitive factors. Since we intended to use the larger and more diverse dataset of the dogs’ first test session for the current and later analyses, we investigated if the best-fitting structure we found in the above analysis would also fit the dataset of the first test sessions. Next, we derived components from the exploration test, box rustle test, and novel object recognition test using PCAs with the same setup as in the cases of the cognitive tasks. In the third step, we introduced these components into the cognitive structure one by one and linked them to the factor(s) highest in the hierarchy. We investigated the modification indexes to determine if adding any additional path would improve the model fit and checked if the regressions among the cognitive tasks and latent variables weakened by introducing these factors. A larger drop in the regression weight would mean that a significant proportion of the variance a given task shares with the others is related to the introduced variable.

We also analyzed if the extracted cognitive factors correlate with an independent measure of the discrimination and reversal learning ability of the dogs. In the latter tests, we coded only the number of trials required to learn the initial association between the stimuli and reward and the number of trials required to learn the reversed association. Since only *N* = 59 dogs participated in the discrimination and reversal learning tasks, which were too low for a SEM, we used Pearson correlation for this analysis.

#### Investigating the existence of distinct age-related influences on the cognitive measures

The number of distinct age-related influences on the different cognitive measures was examined using a series of structural equation models. The initial model, known as the full independence model, introduced age into the structural model but did not link it to any cognitive measure. In subsequent models, age-related effects were analyzed following a broad-to-narrow strategy. This strategy started by adding an age effect to the cognitive factor at the highest level in the hierarchy and then proceeded to successively add additional age effects on lower levels in a stepwise manner, similar to the analytic strategy described in previous literature [24]. We compared the models using the same fit statistics as in the CFA.

At each level of the hierarchy, we kept only those effects that improved the model fit, at least on a trend level, based on the Chi^2^ difference relative to the difference in the degrees of freedom. Once the best-fitting model was identified, we also investigated if omitting any of the higher level effects would improve the model fit further.

#### Data reduction in the questionnaire

In subsequent analyses, we explored the age trajectory of the extracted common factor and its possible modifying effects on both cross-sectional and longitudinal data.

Several questionnaire variables co-varied and, therefore could not be considered independent measures. First, we applied a PCA to separate parts of the questionnaire to obtain composite measures. We conducted these PCAs on a larger sample of questionnaire answers (*N* = 1532) to ensure higher reliability.

In the first PCA analysis, we included 19 demographic variables. Since they were either nominal or ordinal scales, we used a heterogeneous correlation matrix (“hector” function of “polycor” package [78], R statistical software, version 3.6.3 [79] in Rstudio [80]) as the basis of the PCA. We determined the number of potential components using parallel analysis (“fa.parallel” function of “psych” package [81]). Subsequently, we cleaned the PC model by step-by-step elimination of single-component items and items with low loading (< 0.4). From the resulting 5-component structure, only the first three components had acceptable internal consistency, and thus were kept for later analyses: training level (standardized Cronbach’s alpha: 0.723), health issues (standardized Cronbach’s alpha: 0.620), and family (standardized Cronbach’s alpha: 0.533). The component scores were calculated as item averages. Variables that fell out of the PCA were investigated as individual items. Aside from the demographic part, we analyzed three more sub-questionnaires with separate PCAs, following the same methodology as described above.

The set of ten questions about the owners’ attitude towards dogs formed two components: emotional attitude (standardized Cronbach’s alpha: 0.710) and doggy lifestyle (standardized Cronbach’s alpha: 0.660). Cognition and communication-related items (30 items) formed a three-component structure: communication (standardized Cronbach’s alpha 0.858), signs of decline (standardized Cronbach’s alpha: 0.769), and uncontrollability (standardized Cronbach’s alpha: 0.620). Finally, the 11 items about the dogs’ character formed a three-component structure, but only the first had acceptable internal consistency. Thus, we kept only this scale, which we labeled as sociability/trainability (standardized Cronbach’s alpha: 0.720). All PCA results can be found in Table S2 in SI2.

#### Cross-sectional analysis of the age association of the common factor and its modifying factors

Given the significant deviations from normal distribution observed in both the common factor and age (common factor score—D’Agostino test: skew =  − 0.701, *z* =  − 3.126, *p* = 0.002; Anscombe-Glynn test: kurt = 3.099, *z* = 0.524, *p* = 0.600; age—D’Agostino test: skew =  − 0.307, *z* =  − 1.473, *p* = 0.141; Anscombe-Glynn test: kurt = 1.907, *z* =  − 5.462, *p* < 0.001; analyzed with the “moments” package [82]), we opted for robust correlation analysis (“ggscatterplot” function of “ggstatsplot” package [83]) as it is not sensitive to skewness and kurtosis deviations of the variable. Additionally, power transformation of the common factor score was applied to normalize it using Box-Cox analysis (“boxcox” function of “MASS” package [84]) in further analyses.

Subsequently, we explored the modifying effects of owner-reported demographic factors, dog-keeping practices, personality traits, behavior scales, and owner attitudes on the common factor and its relationship with age. To prevent overparameterization, we employed regularized general linear models (lm) with the elastic net approach to identify important factors and covariates associated with the common factor.

The model comprised individual features somewhat independent of the owner and the keeping conditions, including sex (male, female), reproductive status (intact, neutered), breed group (purebred, mixed breed), weight (continuous), height at withers (continuous), body condition (underweight, normal, overweight), training level (first component of the demographic factors), health issues (second component of the demographic factors), and previous experienced trauma (yes, no). Additionally, it encompassed keeping conditions such as time spent playing with the owner per day (< 1 h, > 1 h), off-leash activity per day (< 1 h, 1–3 h, > 3 h), time spent alone per day (none, 1–2 h, 3–8 h, > 8 h), other dogs in the household (none, one, more), owner’s age (18–29, 30–39, 40–49, 50 < years), and family (third component of the demographic factors). Furthermore, the model included the dog personality (DPQ) scores: fearfulness, aggression towards people, activity/excitability, responsiveness to training, and aggression towards animals, as well as the dog behavior and owner attitude scales extracted from the questionnaire items using PCAs: owner’s emotional attitude, owner’s doggy lifestyle, communication, signs of decline, uncontrollability, and sociability/trainability. Interactions of all these parameters with dog age were also incorporated. All parameters were transformed to numeric and then standardized (mean-centered and scaled) for subsequent analysis.

First, we identified the optimal alpha value (0.1) using the elastic net approach with tenfold cross-validation (“train” function of “caret” package, [85]). In a second step, to identify the most important model terms, we ran a second model with tenfold cross-validation using this alpha value (“cv.glmnet” function of “glmnet” package [86]) 1000 times. We considered terms to be important if they appeared in at least 95% of the models with a coefficient above zero. For post hoc comparisons, we used simple slopes analysis (“interactions” package [87]).

#### Estimation of the reliability of the cognitive measures over time

The aim of this analysis was to assess the reliability of the higher level cognitive measures over increasing time periods. Since we tested the dogs in waves, with periods of intensive testing followed by breaks, we created five groups of the retest data based on how much time elapsed since the first (baseline) test session: 0.2 to 0.5 years (*N* = 71); 0.6 to 1.5 years (*N* = 29); 1.6 to 2.0 years (*N* = 44); 2.1 to 2.5 years (*N* = 21); and 2.6 to 3.0 years (N = 22). The reliability was investigated using Intraclass correlation (ICC, two-way mixed model).

#### Longitudinal assessment of the change in the common factor

Finally, we examined the change in the common factor over (up to) 3 years. For this, we calculated the change in the value of the common factor since the baseline measurement. A linear mixed-effects model (“lme4” package [88]) was used to analyze the potential impact of the baseline common factor, the time elapsed since the baseline measurement, as well as the explanatory factors previously included in the cross-sectional analysis on the change in the common factor. Subject ID was included as a random factor. For post hoc comparisons, we used simple slopes analysis (“interactions” package [87]). First, we made basic models, which included only one explanatory factor. Then, we used bottom-up model selection to find the best complex model (“anova” function of “stats” package), where the inclusion criteria were a significant likelihood ratio test for each tested variable.

## Results

### Correlation structure among the cognitive tasks

The parallel analysis conducted on the six cognitive components suggested two factors to be retained in both the raw data and age-residuals of the test data. The KMO measure of sampling adequacy was 0.636 and 0.609 on the two datasets, respectively, and Bartlett’s test of sphericity was highly significant (*p* < 0.001), confirming that the sample size was adequate for the EFA.

The unrotated EFA revealed that five components loaded on the first factor (Table S4 in SI2), which we termed the common factor. The results were the same when the analysis was performed on the age-residuals of the data. The two components that did not load significantly (> 0.3) on this factor were flexibility and attention to object. If these two were removed from the analysis, the first factor explained 30.0% of the common variance in the raw data and 28.1% in the age residuals (Table S5 in SI2).

In the rotated analysis (Table 1), the KMO measure of sampling adequacy was adequate for both datasets (0.695; 0.677, respectively), and Bartlett’s test of sphericity was highly significant (*p* < 0.001). The parallel analysis suggested two factors to be retained, which together explained 41.1% of the common variance in the raw data and 37.9% in the age residuals. Performance on three tasks (manipulative persistency, problem solving, and memory) loaded on factor 1, which we termed as Individual problem solving. Higher scores indicate greater persistence in searching for hidden food, faster success in obtaining it, and better memory in recalling where it was hidden. The two tasks that formed the second factor (clicker game and training for eye contact) are related to associative learning; thus, we termed this factor as learning. Higher scores mean faster learning of the association between establishing eye contact with the experimenter or any performed behaviors and a reward. The two factors correlated positively with each other (raw data, *r* = 0.466, age residuals, *r* = 0.434), suggesting a shared common source of variance.

**Table 1:** Pattern matrix of the exploratory factor analysis conducted with oblimin rotation on the raw data and the (linear) age-residuals of the test data. Loadings > 0.3 are in bold

Cognitive component	Raw data	Age-residuals		
	Individual problem solving	Learning	Individual problem solving	Learning
Persistency	**0.747**	− 0.122	**0.733**	− 0.126
One-trial learning	0.077	**0.389**	0.053	**0.456**
Problem solving success	**0.680**	0.105	**0.680**	0.115
Associative learning	− 0.058	**0.751**	− 0.031	**0.631**
Memory	**0.438**	0.199	**0.461**	0.102
Eigenvalue	2.140	1.031	2.039	1.077
Cronbach’s alpha	0.674	0.477	0.664	0.460

**Fig. 1:**
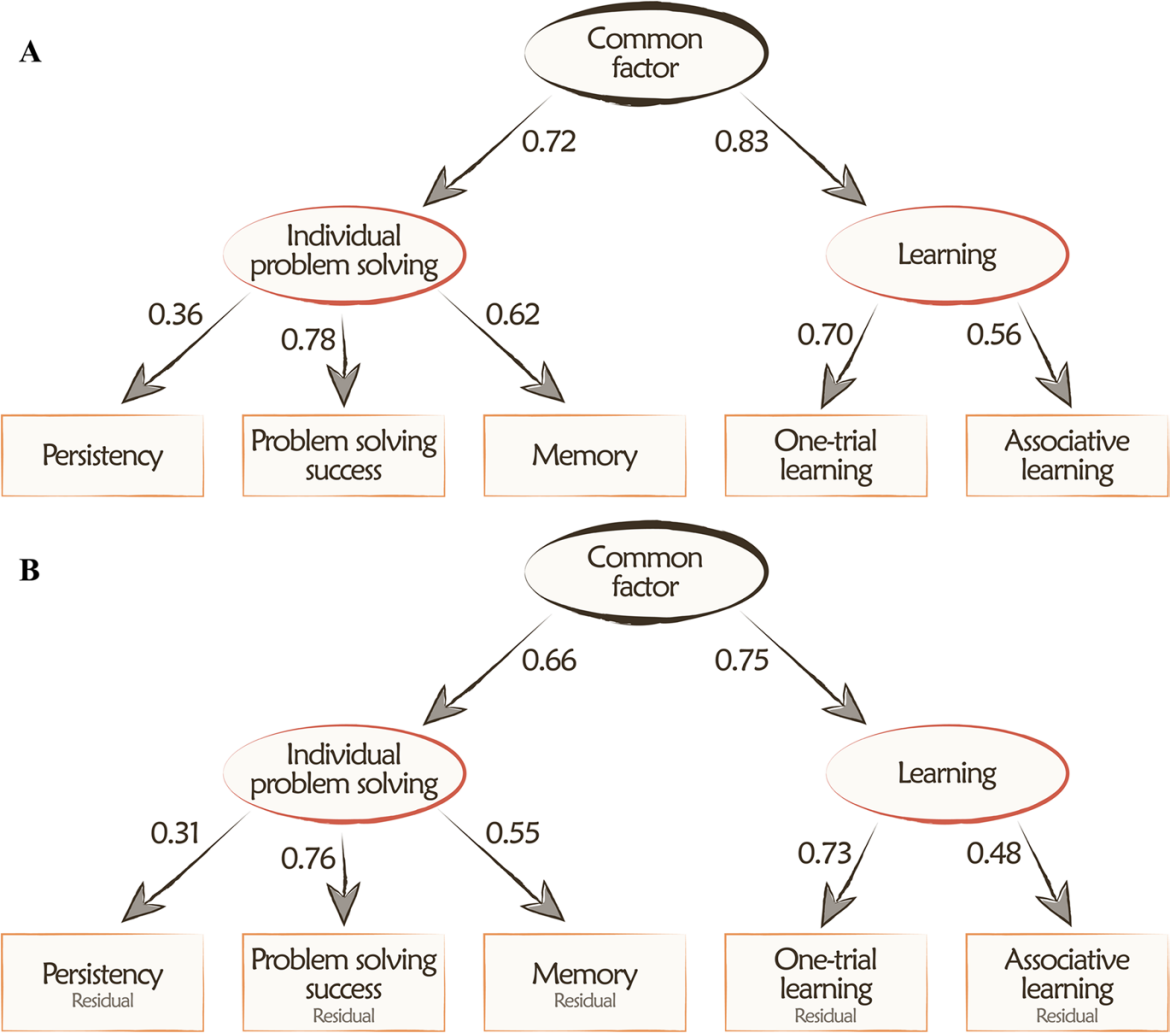
The Hierarchical *g* factor model (confirmatory factor analysis) results on the raw test data (**A**); and the age-residuals of test data (**B**) (*N* = 72). The five cognitive components were entered as observed variables (indicators) and are represented by rectangles. Individual problem-solving, Learning, and the common factor were entered as latent factors and are represented by ovals. Arrows from oval to rectangle indicate regressions, and values associated with each path are standardized regression coefficients

We utilized three alternative model structures from those described in Arden and Adams [44] and fitted them onto the data (refer to Figure S1 in SI3). The “Hierarchical *g* model” assumed the existence of both the separate cognitive domains (first-order factors) and a common factor. The “No *g* model” posited the existence of separate cognitive domains but not the common factor, implying independence among the first-order latent factors. Conversely, the “*g*-only model” presumed the existence of the common factor but not the separate cognitive domains. The CFA analysis revealed that in both the raw data and the age residuals of the test data, the best model fit was achieved with the hierarchical *g* model (see Table 2 and Fig. 1). This model version also demonstrated a significantly better model fit compared to any other models (smallest Chi^2^ difference = 5.30 with df difference of 1), confirming the existence of both the two cognitive domains and the common factor itself. For further analyses, we computed the factor scores of the two cognitive domains as the average of the related task performances, and the score for the common factor as the average of the two cognitive domain scores.

**Table 2:** Model fit indexes of the three model structures on the raw test data and the age-residuals (*N* = 72). The best-fitting model (hierarchical *g*) was also fitted to the first test session data (*N* = 124) of the animals, and additional validation analyses were run on this sample where activity (from the exploration test), Following (from box rustle test), or preference for novelty (from novel object recognition test) was added to the structural model

Models (raw data)	Chi^2^	df	RMSEA (90% CI)	PPP	AIC	BIC
Hierarchical *g* model	1.565	4	0.027 (0.000–0.110)	0.859	23.565	48.608
No *g* model	12.594	6	0.124 (0.001–0.221)	0.097	30.594	51.084
*g*-only model	6.857	5	0.072 (0.000–0.191)	0.325	26.857	49.624
Models (age-residuals)						
Hierarchical *g* model	1.516	4	0.000 (0.000–0.107)	0.866	23.516	48.560
No *g* model	8.466	6	0.076 (0.000–0.184)	0.304	26.466	46.956
*g*-only model	6.984	5	0.075 (0.000–0.193)	0.314	26.984	49.751
Hierarchical *g* model	4.365	4	0.027 (0.000–0.141)	0.510	26.365	57.388
Hierarchical *g* model + activity	4.950	8	0.000 (0.000–0.074)	0.880	30.950	67.296
Hierarchical *g* model + following	7.517	8	0.000 (0.000–0.102)	0.681	33.517	69.969
Hierarchical *g* model + preference for novelty	7.177	8	0.000 (0.000–0.099)	0.710	33.170	69.629

### External validation of the cognitive factors

**Fig. 2:**
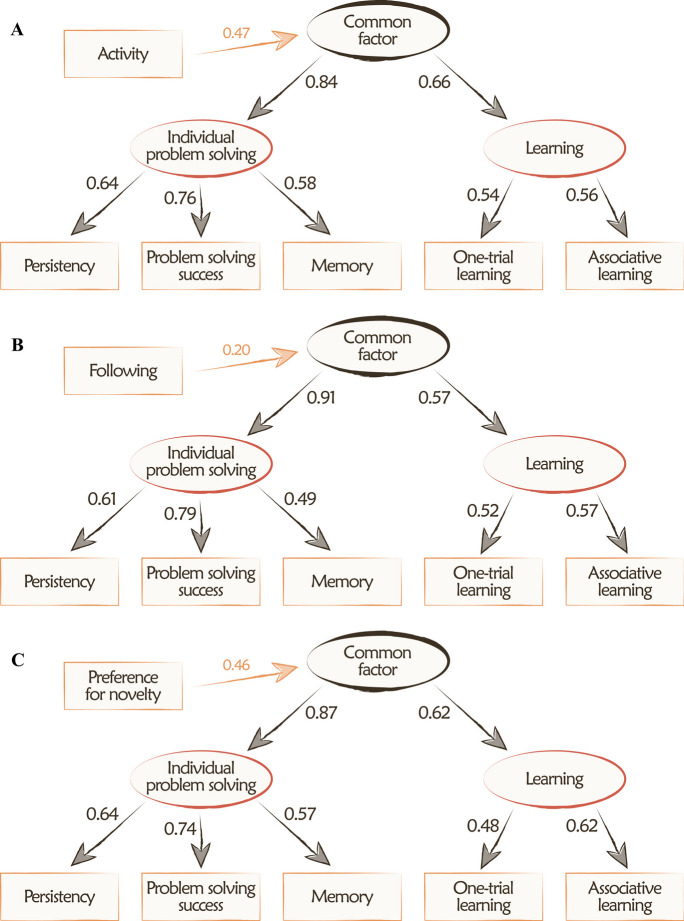
Structural equation models used for the external validation of the common factor (*N* = 129). The hierarchical model of the cognitive tasks was added three additional variables (separately): **A** the Activity component extracted from the exploration test; **B** the following component extracted from the box rustle test; **C** the preference for novelty component extracted from the novel object recognition test. Arrows indicate regressions, and values associated with each path are standardized regression coefficients

Activity during the exploration test and the preference for novelty component of the novel object recognition test exhibited moderate positive relationships, while following in the box rustle test showed no significant association with the common factor (standardized regression coefficients = 0.473, 0.462, and 0.202, respectively; Fig. 2). All three models with the added variable demonstrated good fits to the data (Table 2), and modification indexes indicated that no additional regressions would improve the model fit.

Regarding the discrimination and reversal learning test, the number of trials required to learn the initial association between the stimuli and reward correlated negatively with the common factor (*r* =  − 0.418, *p* = 0.001), and weaker but still significant correlations were found with both cognitive domains (individual problem solving: *r* =  − 0.330, *p* = 0.011; learning: *r* =  − 0.353, *p* = 0.006). The number of trials required to learn the reversed association correlated negatively with the common factor (*r* =  − 0.337, *p* = 0.009) and individual problem-solving (*r* =  − 0.328, *p* = 0.011). In both cases, dogs with higher common factor scores required fewer trials to learn the initial or reversal association.

### Investigating the existence of distinct age-related influences on the cognitive measures

**Fig. 3:**
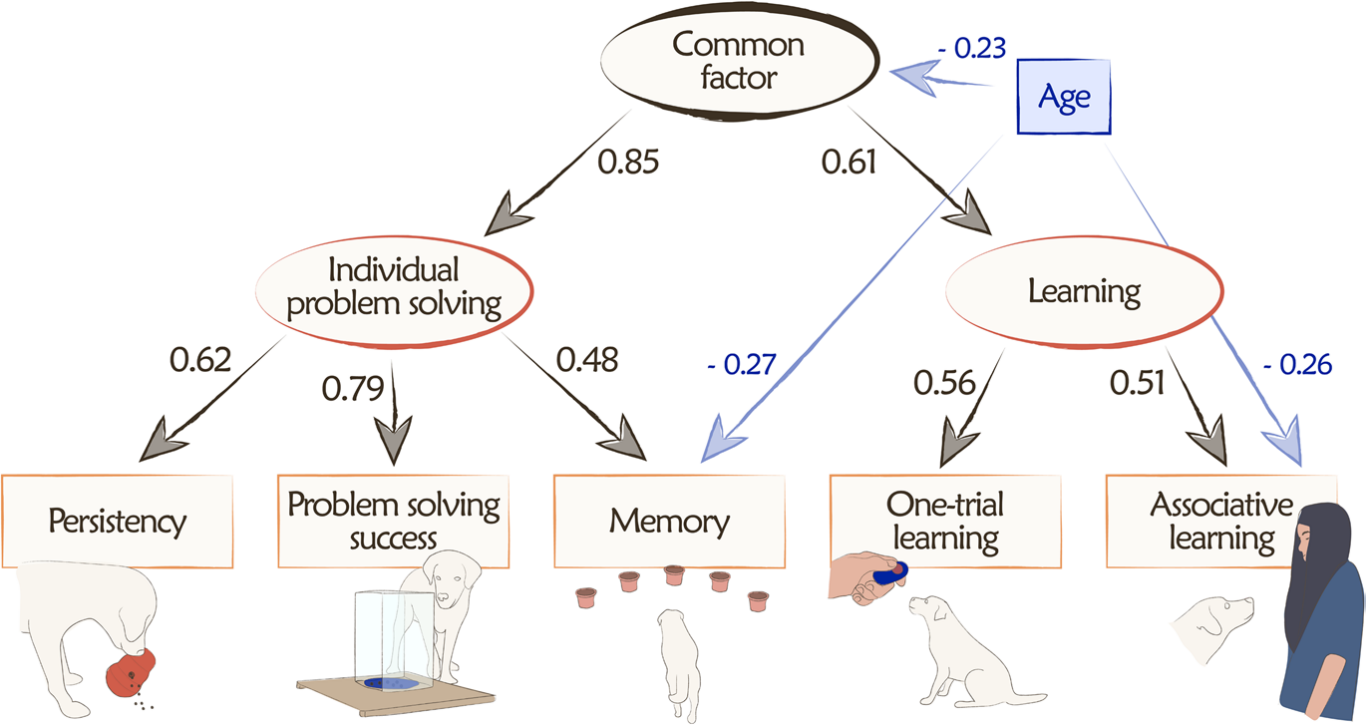
The best-fitting structural equation model depicting the statistically distinct age effects on the dogs’ cognitive measures on different levels of hierarchy (*N* = 129). Arrows indicate regressions, and values associated with each path are standardized regression coefficients

The initial, full independence model (M0) was the hierarchical model with age included but having no association with any cognitive measure. This model exhibited a very poor fit to the data (Table 3). In the first-level model (M1), the sole age-related effect added to the model was the common factor (i.e., at the highest, second-order level in the hierarchical structure). Although this model still significantly deviated from the data, adding this regression significantly improved the model fit and was retained for subsequent models. The second-level model (M2) permitted an additional direct path from age to one of the first-order factors (as there were only two first-order factors, the result was the same regardless of which). This path improved the model fit on a trend level compared to the common-only model (M1), thus it was also retained. Third-level models (M3.1–3.5) added an extra path from age to each of the raw task performances, in addition to the first-order and common factor. According to the statistics, adding a path to memory significantly improved the model fit, while the path to problem-solving success and association learning improved it on a trend level. After adding a path to memory (in addition to the paths to the first-order and common factor, M4.1–2), an additional path to problem-solving success no longer improved the model fit, while a path to association learning did, so this latter was retained. In the final step, we explored if we could improve model fit by removing age effects on higher levels. Examination of the parameter estimates of the best-fitting model suggested that the path to the first-order factor was no longer significant (standardized regression weight = 0.035), so it was removed from the model. The model fit did not substantially decrease with this change. However, upon removing the path to the common factor, the model fit decreased on a trend level, so this path was retained. Thus, the final model (M5.1) contained paths to the common factor, memory, and associative learning and exhibited an excellent fit to the data. The path diagram of this final model and its standardized regression coefficients are displayed in Fig. 3.

**Table 3:** Model fit indexes and comparisons of the series of structural equation models investigating different combinations of age effects on the cognitive measures. The best-fitting model (M5.1) is in bold

Model	Chi^2^	df	RMSEA (90% CI)	PPP	AIC	BIC	ΔChi^2^/Δdf
M0: Age to none (full independence model)	30.389	9	0.139 (0.87–0.195)	0.004	54.389	88.233	-
M1: Age to common	18.370	8	0.103 (0.040–0.165)	0.076	44.370	81.034	12.019/1, *p* < 0.001 (vs. M0)
M2: Age to common and first-order	15.456	7	0.099 (0.029–0.167)	0.102	43.456	82.940	2.914/1, *p* < 0.1 (vs. M1)
M3.1: Age to common, first-order, and persistence	13.757	6	0.103 (0.028–0.175)	0.100	43.757	86.061	1.699/1, *p* > 0.1 (vs. M2)
M3.2: Age to common, first-order, and problem-solving success	11.728	6	0.088 (0.000–0.163)	0.172	41.728	84.033	3.728/1, *p* < 0.1 (vs. M2)
M3.3: Age to common, first-order, and memory	5.121	6	0.000 (0.000–0.107)	0.696	35.121	77.425	10.335/1, *p* < 0.001 (vs. M2)
M3.4/5: Age to common, first-order, and one-trial learning/associative learning^a^	12.731	6	0.096 (0.010–0.169)	0.133	42.731	85.036	2.725/1, *p* < 0.1 (vs. M2)
M4.1: Age to common, first-order, memory, and problem-solving success	5.119	5	0.014 (0.000–0.127)	0.569	37.119	82.243	0.002/1, *p* > 0.1 (vs. M3.3)
M4.2: Age to common, first-order, memory, and associative learning	2.249	5	0.000 (0.000–0.077)	0.892	34.249	79.374	2.872/1, *p* < 0.1 (vs. M3.3)
M5.1: Age to common, memory, and associative learning	**2.269**	**6**	**0.000 (0.000–0.053)**	**0.946**	**32.269**	**74.573**	** − 0.020/ − 1, *p* > 0.1 (vs. M4.2)**
M5.2: Age to memory and associative learning	5.627	7	0.000 (0.000–0.097)	0.753	33.627	73.111	− 3.358/ − 1, *p* < 0.1 (vs. M5.1)

^a^There were only two tasks related to the learning factor, so the results were the same no matter which was linked to age. However, as associative learning had a stronger correlation with age, we kept that path in the model

### Cross-sectional analysis of the age association of the common factor and its modifying factors

**Fig. 4:**
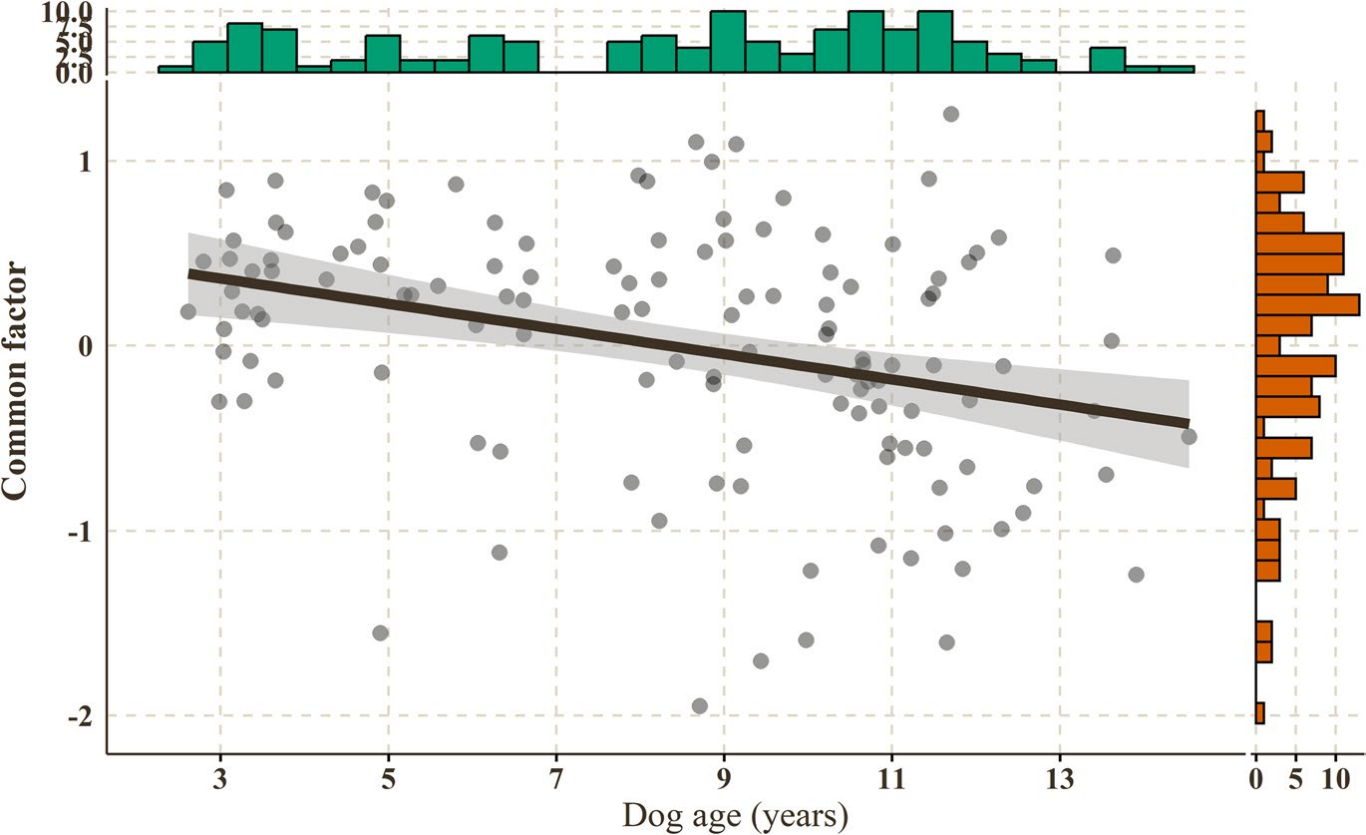
The negative correlation between age and the common factor score (Winsorized robust correlation, *N* = 128)

The common factor score, as expected from the SEM results, correlated negatively with the animals’ age (winsorized robust correlation: *r*_*w*_[CI_95%_] =  − 0.39[− 0.53– − 0.23], *t*(126) =  − 4.75, *p* < 0.001; Fig. 4).

**Fig. 5:**
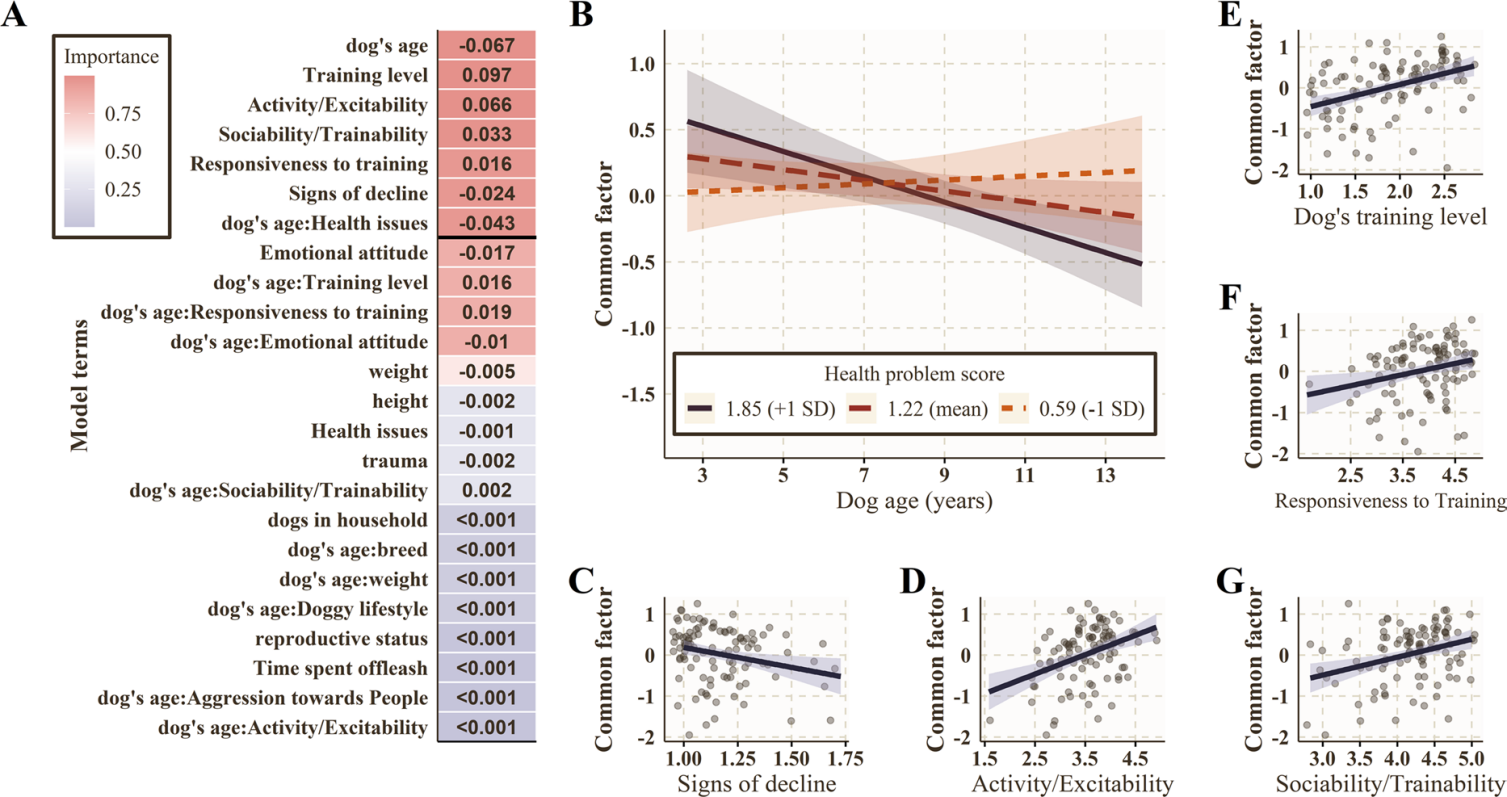
Results of the regularized general linear models (lm) with elastic net approach on the cross-sectional data (*N* = 121). **A** The mean coefficients (numbers in cells) were obtained from the 1000 cross-validation models based on the elastic net analysis. Effects with zero coefficients appearing in all models are omitted for visibility. The color scale shows the terms’ relative importance (ratio of their appearance with above zero coefficient in the models). The horizontal black line indicates the 95% threshold. **B**, **C**, **D**, **E**, F, **G** The main effects of different covariates on the common factor and its age association based on the cross-sectional data. **B** Health issues strengthened the negative association between the common factor and age (black line: + 1 SD, red line: mean value, orange line: − 1 SD of health issues). **C** Owner-reported signs of decline had a negative association with the common factor. The common factor was positively associated with **D** the dogs’ activity/excitability trait, **E** training level, **F** responsiveness to training trait, and also **G** their sociability/trainability score. In these latter five graphs, the fitted lines show the estimated main effects, the shaded areas represent 95% confidence intervals around the fits, and the dots represent the individual values as partial residuals

Based on the regularized linear model for demographic features, keeping conditions, personality, and behavioral scales, we identified five important main effects and one interaction term (Fig. 5A).

We found that overall health moderated the negative relationship between aging and the common factor (Fig. 5B). The simple slopes analysis revealed that the negative association between dogs’ age and their common factor score was significant only if the dogs’ health issue score was above 1.121, and the age association was more negative if the health issue score was higher (at 0.62 (− 1 SD) health issue score: ß ± SD = 0.01 ± 0.02; *t* = 0.26; *p* = 0.80; at 1.24 (mean) health issue score: ß ± SD =  − 0.05 ± 0.02; *t* =  − 2.56; *p* = 0.01; at 1.87 (+ 1 SD) health issue score: ß ± SD =  − 0.10 ± 0.03; *t* =  − 3.64; *p* < 0.001). Additionally, independent from age, the common factor was negatively associated with the owner-reported signs of decline (Fig. 5C): dogs with a higher prevalence of cognitive dysfunction symptoms had lower common factor scores. The common factor was positively associated with the dogs’ activity/excitability trait (Fig. 5D), training level (Fig. 5E), responsiveness to training trait (Fig. 5F), and sociability/trainability score (Fig. 5G): more trained, more active, sociable, and easier to excite dogs had higher common factor scores.

### Estimation of the reliability of the cognitive measures over time

We found that the reliability of the common factor declines over time, yet it becomes unreliable only after approximately 2.5 years (Table 4). Regarding the first-order factors, individual problem-solving’s reliability demonstrates a faster decline over time, already indicating poor temporal consistency after 1.5 years (although that drop could be due to the low sample size), and the reliability diminishes to negligible after 2.5 years. In contrast, the reliability of the learning factor exhibits a gradual decline, remaining reliable even after 2.5 years.

**Table 4:** Estimates of the reliability of the common factor and the two first-order cognitive domains across different time periods

Time since the first test	ICC (95% CI)	*F*	*p*-value
Common factor			
≤ 0.5 years (*N* = 72)	0.909 (0.853–0.943)	10.933	< 0.001
> 0.5– < 1.5 years (*N* = 30)	0.825 (0.627–0.918)	5.709	< 0.001
> 1.5– < 2 years (*N* = 45)	0.719 (0.486–0.847)	3.563	< 0.001
> 2– < 2.5 years (*N* = 2 1)	0.869 (0.676–0.947)	7.607	< 0.001
> 2.5– < 3 years (*N* = 22)	0.282 (0–0.702)	1.392	0.227
Individual problem solving			
≤ 0.5 years (*N* = 72)	0.810 (0.696–0.881)	5.263	< 0.001
> 0.5– < 1.5 years (*N* = 30)	0.703 (0.377–0.859)	3.372	< 0.001
> 1.5– < 2 years (*N* = 45)	0.478 (0.049–0.713)	1.914	0.017
> 2– < 2.5 years (*N* = 21)	0.788 (0.477–0.914)	4.710	< 0.001
> 2.5– < 3 years (*N* = 22)	0.094 (0–0.624)	1.104	0.412
Learning			
≤ 0.5 years (*N* = 72)	0.874 (0.798–0.921)	7.929	< 0.001
> 0.5– < 1.5 years (*N* = 30)	0.798 (0.570–0.905)	4.959	< 0.001
> 1.5– < 2 years (*N* = 45)	0.801 (0.636–0.892)	5.033	< 0.001
> 2– < 2.5 years (*N* = 21)	0.724 (0.319–0.888)	3.617	0.003
> 2.5– < 3 years (*N* = 22)	0.606 (0.051–0.836)	2.537	0.019

### Longitudinal assessment of the change in the common factor

**Fig. 6:**
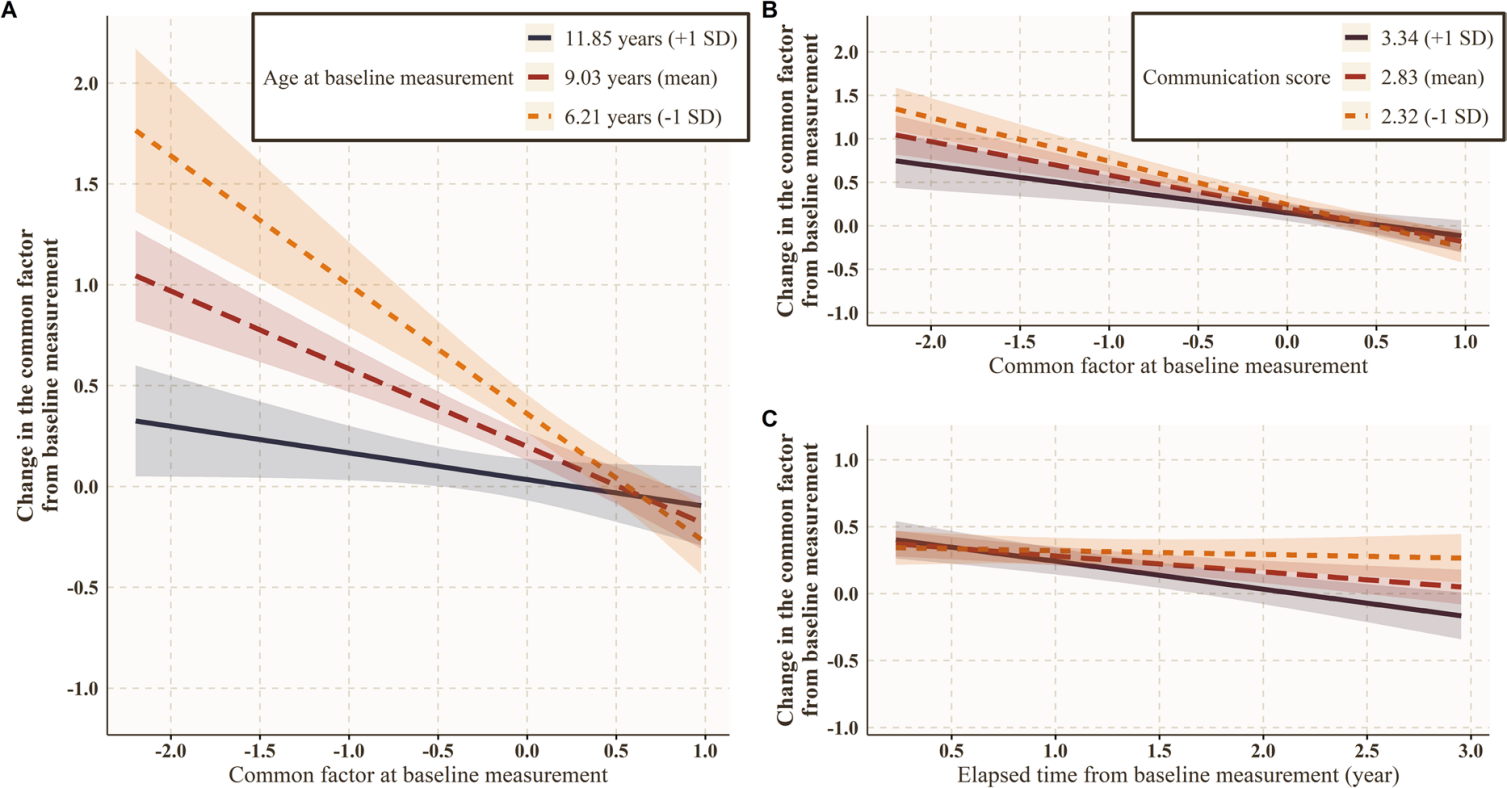
Results of the linear mixed-effects model on the longitudinal data (*N* = 187). **A** Interaction between dogs’ baseline age and baseline common factor score (black line: + 1 SD, red line: mean value, orange line: − 1 SD of age). **B** Interaction between dogs’ communication score and their baseline common factor score, and **C** the time elapsed since baseline measurement (black line: + 1 SD, red line: mean value, orange line: − 1 SD of communication score)

The change in the common factor from the baseline measurement was influenced by dogs’ baseline age, baseline common factor score, and baseline communication score, as well as the time elapsed since the baseline measurement. We observed an interaction between dogs’ baseline age and baseline common factor (*p* < 0.001). In younger dogs, the association was more negative between the change in the common factor and baseline common factor (at age 6.21 years (− 1 SD): ß ± SD =  − 0.64 ± 0.09; *t* =  − 7.44; *p* < 0.001; at age 9.03 years (mean): ß ± SD =  − 0.38 ± 0.05; *t* =  − 7.48; *p* < 0.001; at age 11.85 years (+ 1 SD): ß ± SD =  − 0.13 ± 0.07; *t* =  − 1.88; *p* = 0.060; Fig. 6A). The dogs’ communication score was in interaction with both their baseline common factor (*p* = 0.008) and the time elapsed since baseline measurement (*p* = 0.005). The association was more negative between the change in the common factor and baseline common factor in less communicative dogs (at 2.32 (− 1 SD) communication score: ß ± SD =  − 0.50 ± 0.06; *t* =  − 8.21; *p* < 0.001; at 2.83 (mean) communication score: ß ± SD =  − 0.38 ± 0.05; *t* = -7.48; *p* < 0.001; at 3.34 (+ 1 SD) communication score: ß ± SD =  − 0.27 ± 0.07; *t* =  − 3.77; *p* < 0.001; Fig. 6B). Meanwhile, in more communicative dogs, the association was more negative between the change in the common factor and the time elapsed since baseline measurement (at 2.32 (− 1 SD) communication score: ß ± SD =  − 0.03 ± 0.04; *t* =  − 0.62; *p* = 0.53; at 2.83 (mean) communication score: ß ± SD =  − 0.12 ± 0.03; *t* =  − 3.61; *p* < 0.001; at 3.34 (+ 1 SD) communication score: ß ± SD =  − 0.21 ± 0.05; t =  − 4.44; *p* < 0.001; Fig. 6C).

## Discussion

The primary objective of this study was to investigate previously unexplored aspects of canine cognitive aging and conduct a comprehensive analysis of age-related changes in canine cognitive measures using both cross-sectional and longitudinal data. This included investigating the number of statistically independent age effects that act upon the cognitive structure of dogs, as well as examining how individual and environmental characteristics may modify these age effects.

### Correlation structure among the cognitive tasks

We first examined the structure underlying individual differences in a selection of cognitive tasks to investigate this. Our findings revealed consistent individual differences in the dogs’ cognitive performance across tasks and over time. Specifically, performance in five tasks correlated positively with one another, forming a positive manifold. Approximately one-third of the variance in the aggregate performance on these tasks could be attributed to a single underlying common factor. Confirmatory factor analysis confirmed the presence of this common factor but also indicated that some tasks had additional sources of shared variance not accounted for by the common factor. Our analyses indicated the presence of two domain-specific latent factors at an intermediate level of the hierarchy. We labeled one individual problem-solving as it was related to three tasks where food could be obtained by working independently from humans, and the other learning, as it was composed of two tasks where higher performance indicated faster associative learning capacity. The best-fitting hierarchical structure we found was in harmony with the findings of [44] but contradicted those of [34, 36, 37]. These latter studies suggested that multiple independent factors explained the individual variation in cognition better, which did not fit our dataset, where the weakest model fit was found when only first-order latent factors were postulated without a common factor linking them.

Our study demonstrates several improvements in sample selection, methods, and the rigor of the statistical procedures compared to earlier attempts. Firstly, the range, diversity, and familiarity with the tests within a battery are crucial for effectively separating the effects of *g* from task-specific contributions to performance [45]. The cognitive battery used in this study explored a broader range of tests compared to some earlier efforts ([33, 44] but see [34, 36]), with tasks that were somewhat similar to those found in relation to *g* in other animal species [45, 74]. These tasks included problem-solving (evaluating persistence, speed, and success in finding hidden food), associative learning, and memory. Regarding memory, most human and non-human studies emphasize that working memory is a key component of the general intelligence factor [89]. However, less evidence is available for short-term memory [45, 90]. In our memory task, although the dogs were distracted between the storage of information and the recall phase, this intervention may not have been enough to classify the task as measuring working memory. Nevertheless, the positive relationship between better performance in the memory task and all other tasks included in the analysis aligns well with the notion that improved storage and recall of information are fundamental aspects of cognition in dogs as well.

Another important limitation of earlier studies is that they rarely demonstrated consistency in cognitive ability across contexts or time, so the results of these studies may refer only to correlations between snapshots of momentary states [65]. Moreover, the potential low reliability of some tasks may have weakened their correlations with other tasks [45]. In our study, we investigated both the short-term repeatability of the individual tasks and the long-term temporal consistency of the extracted latent factors. Regarding the former, we found that the pointing and the attention tasks were not repeatable. The finding regarding pointing aligns with [44], which also found a low level of repeatability in point-following success (although they still included that task in their analyses). Regarding attention, although this task has been previously used to assess (sustained) attention in dogs [1, 7], it is possible that the variables we coded in this task were only partly related to attention and were more affected by the dogs’ propensity to orient towards the owner during uncertainty. In mice, attention, especially selective attention, was deemed an important component of the general cognitive factor and was suggested as a candidate process that mediates the relationship between working memory and general intelligence [89–92]. Therefore, a more accurate measurement of attention is necessary to determine whether this capacity correlates with other cognitive performances in dogs, as well.

Regarding the long-term temporal consistency of the latent factors, we demonstrated that all factors exhibit decreasing reliability over time, yet they remain reliable 2.5 years after the initial measurement. However, beyond 2.5 years, the reliability diminishes for both the individual problem-solving and, likely consequently, the common factor as well. In contrast, the learning factor remains consistent even after 3 years, suggesting that the two cognitive domains possess somewhat different internal and/or environmental correlates. This level of consistency is relatively low compared to the temporal consistency of human IQ (as reviewed in [93]), and even lower than other composite traits in dogs, such as personality (which has been found to be consistent for at least up to four years [47, 94]). A potential explanation for this discrepancy could be the generally old age of the current sample population, with 40% of the dogs already in their geriatric phase (over 10 years of age) at their first test session. The higher inter-individual variation in aging success within this population may introduce a random effect on the aging curve of cognition, consequently leading to generally lower levels of consistency over time.

Another methodological improvement in the current study was to assess not only the reliability but also the validity of the extracted cognitive measures. The common factor we identified is presently only a statistical construct representing the shared variance of the first-order constructs; it requires validation before it can be interpreted as a candidate for *g* (general intelligence) factor [57].

The first type of external validation involved linking the latent common factor to individual characteristics theoretically related to *g* in humans and non-human animals, for which we chose exploration tendencies and neophilia. High levels of exploration and neophilia have been linked to better problem-solving success and learning speed across various species (as reviewed in [63–66]). These behaviors are close to the openness to experience personality trait in humans, which has the strongest link to IQ among the Big Five traits [95]. However, the relationship between these characteristics and cognitive performance, including *g*, is still a subject of inquiry [57, 96]. On the one hand, exploration might causally promote general cognitive abilities. Higher exploratory tendencies and lower neophobia could lead to more learning and problem-solving opportunities throughout life, and this cumulative experience effect can improve the animals’ general abilities over time [74, 97, 98]. On the other hand, actual performance in cognitive tasks may depend on the dogs’ motivation and persistence in engaging with these tasks, which can be linked to their personality traits (e.g., [99, 100]). Animals with higher exploratory tendencies and lower neophobia may appear to be better at cognitive tasks simply because they are more motivated to engage in them or manipulate the apparatus, even if their individual skill level in that particular cognitive ability is not different from that of more fearful or neophobic individuals [67]. Sih and Giudice [64] proposed an integrated framework highlighting a trade-off between speed and accuracy in cognitive performance. Higher boldness, proactive, and exploratory tendencies are suggested to be associated with a “fast” cognitive style, making quicker but less accurate decisions than individuals with a “slow” cognitive style. However, a more recent meta-analysis by [66] failed to find general support for this hypothesis, suggesting it requires refinement. An important piece of this puzzle may come from the current study, as we demonstrated that although these characteristics moderately correlate with the latent common cognitive factor, they are not part of the cognitive structure. Introducing these external factors into the model did not weaken the associations between the cognitive tasks and the late and even slightly improved the general model fit. This improvement suggests that these non-cognitive characteristics do not account for a substantial portion of the relations among the cognitive tasks; they represent only external correlates.

The second type of validation of the common factor as a *g* factor candidate involved examining its correlation with an independent measure of abilities known to correlate with *g* in other species. Reversal learning tasks are typically included in cognitive test batteries across various species because they assess fundamental cognitive abilities and are less influenced by species-specific cognitive adaptations [57, 101]. In our study, the discrimination and reversal learning task was not originally part of the test battery, and there was approximately a 2-week gap between the measurements, with only a subset of the subjects participating in both. Despite these constraints, on this smaller sample, we found a moderate correlation between the dogs’ common factor score and their performance in both the discrimination and reversal learning phases. Dogs with higher common factor scores required fewer trials to learn the initial discrimination and the reversed association between the stimuli (location) and reward. This finding provides evidence that our psychometric common factor represents a domain–general cognitive factor.

Furthermore, a parallel investigation utilizing the common factor as a summary variable for cognitive performance (without specific consideration for its comparability with the human *g*) suggests that animals with high factor scores exhibited greater flexibility in learning associations with different cue types (object feature versus location [70]). This finding aligns with human research linking (fluent) intelligence to cognitive flexibility [102, 103].

Taken together, these findings suggest that the common factor we identified shows some similarity in content, structure, and external correlates with the general cognitive factor observed in other species, including humans. Thus, our results lend support to the notion that similar to some rodent, primate, and bird species (see reviewed in [45, 57, 104]), a general cognitive factor also exists in dogs.

### Distinct age-related influences on the cognitive measures

We conducted an additional validation to determine if the correlations among the tasks were influenced by a common confounding factor, namely age. It was plausible that the cognitive tasks correlated with each other not only because they were affected by a common latent cognitive factor, but also because they all decline with age. However, running the analyses on the age residuals of the data did not alter the structure derived from the exploratory and confirmatory factor analyses. This confirmed that the extracted canine *g* factor is not a statistical artifact.

Among the five cognitive tasks, performance in the training for eye contact and memory tasks correlated significantly (and negatively) with age, which is consistent with previous studies [7, 11, 105], while problem-solving had only a trend-level negative age association. The strongest age effect was found in the putative canine *g* itself, which aligns well with findings from both human and animal studies [23, 106, 107]. The question was whether any or all of these tasks have a unique age-related influence or at least some of these age associations found in the individual tasks are due to the same global age-related decline in cognition, reflected in the age effect on the putative canine *g*. Salthouse [23] emphasized that in a hierarchical structure, the effects of age on narrower abilities cannot be investigated unless the effects on higher order factors are first controlled for.

When we included age as an external factor in the hierarchical cognitive structure, the best-fitting model revealed three statistically independent age effects. Two of these effects operated at the level of individual tasks (associative learning and memory), while the third operated at the level of the second-order common factor (putative canine *g*). These results are significant in at least two major respects. Firstly, they demonstrated that the age effects on the two training for eye contact and memory tasks remain significant even after controlling for the age effects at higher levels of the hierarchy. This indicates that they represent unique age-related influences and likely have distinct background mechanisms and may display unique aging dynamics. On the other hand, we also observed that the age effects on the memory and problem-solving tasks were not independent of each other, as entering the age–memory regression in the model nullified the age-problem solving association. This change suggests that these tasks may share a common cause or that memory acts as a mediator for a larger portion of the age effects on problem-solving performance, or vice versa. Due to our small sample size, we were unable to specifically test these latter explanations, leaving them as topics for future studies.

The second major conclusion drawn from our results is the discovery of statistically independent negative age-related influence on the second-order canine *g* factor. Given that the common practice in dog cognitive aging studies is to analyze age effects for each task separately, this is the first time such a general age effect has been reported. This indicates that separate mechanisms are needed to account for not only the task-specific, but also for the general age-related influences in dog cognition. Moreover, it also calls attention to the notion that at least parts of the age-related influences are shared across tasks, so assessing and interpreting aging effects on different tasks independently can lead to pseudoreplication.

In summary, similar to humans, our study demonstrates that there are multiple unique age-related influences on various cognitive tasks. It is noteworthy that two of these influences (memory and the common factor) align with human results [23, 24, 108]. Further investigations could shed light on the specific neurobiological mechanisms behind the individual and shared age effects, thus significantly contributing to human aging studies in this field.

### Cross-sectional analysis of the age association of the canine g and its modifying factors

In the next part of the study, we conducted a wide range of analyses to investigate how individual and environmental characteristics modify the effect of age on cognitive performance in both cross-sectional and longitudinal samples.

In the cross-sectional sample, we discovered that the canine *g*’s age-related decline is associated with the individuals’ health issues, with a stronger negative correlation between age and canine *g* observed in dogs with poorer health status, while no association was found in dogs in good health. These findings align with some human studies [106, 109] but contradict others (e.g., [24]), which demonstrated that controlling for health status did not markedly alter the magnitude of the age-cognition correlations. It is important to note that health problems cannot be entirely isolated from lifestyle factors known to affect cognitive aging. Pre-existing health issues or age-related decline in health could lead to lower levels of physical and mental exercise and social engagement, and the attenuation of these preventive factors could be what accelerates the rate of cognitive decline [110].

Aside from health conditions, no other factors were found to interact with age in affecting canine *g*. However, we observed numerous main effects associated with the dogs’ *g* factor score, providing further external validation for this factor. Activity/excitability, training level, responsiveness to training, and sociability–trainability traits showed positive correlations with the canine *g* score, while the signs of decline component showed a negative association. The signs of decline component comprises symptoms of the canine cognitive dysfunction syndrome (CCD) [9, 111], which shows high phenotypic similarity to cognitive symptoms in aged humans and also shares some physiological characteristics suggesting similar neuropathological pathways to human dementia [112]. Given that cognitive impairment is the main symptom of CCD, it is not surprising that worse general cognitive performance was associated with a higher prevalence of the other CCD symptoms. This association is consistent with human studies, where high (premorbid) IQ was associated with better prognosis and milder symptoms of dementia but had no impact on survival rates [50, 52, 113].

The positive association between the canine *g* and owner-reported activity/excitability trait aligns well with the results of the behavior test regarding the links between canine *g* and exploration and neophilia. Dogs that are more active and open to new experiences may also be easier to train. The positive relationship between training experience and trainability (a personality trait characterized by a dog’s willingness to attend to and obey its owner and low levels of distractibility and resistance to correction [114]) and cognitive performance was expected based on previous studies [1, 10]. Trained dogs were found to spend more time interacting with the task, whereas untrained dogs spent more time looking back at humans [115, 116]. However, the dogs’ trainability does not seem to affect all cognitive tasks uniformly. For example, [117] found no correlation between the trainability assessment of the dogs and the number of training trials needed to pass the criteria in an associative learning task, and [34] also found no effect of lifelong training on more comprehensive cognitive factors. Nevertheless, the positive association between the level of training and canine *g* aligns with the human literature on education and IQ [118]. The robustness of this association is evident from the fact that all three traits measuring trainability or the level of training experience showed it. However, it remains unclear whether training is only an external correlate of canine *g* or whether it explains a measurable portion of the shared variance across cognitive tasks, providing another topic for future research.

### Longitudinal assessment of the change in the canine *g*

In addition to the cross-sectional analysis, we investigated whether and how the canine *g* factor changes over 3 years. The dogs’ age, baseline performance, and communication scores emerged as crucial factors involved in interactions with one another. Younger dogs (around 6 years old) with a low baseline canine *g* score exhibited greater improvement in performance over the years, suggesting higher cognitive plasticity [119]. Their enhanced performance may be attributed to prior test experience, a factor we could not separate from potential systematic cognitive improvement. In contrast, older dogs (around 12 years old) showed a less pronounced experience effect, possibly due to their lower cognitive plasticity, leaving less room for improvement. Intervention therapies were also reported to be more effective when applied to younger animals [120–122], suggesting that the natural age-related decline may be more reversible in younger age, while it may become irreversible after a certain age. Our results underscore the importance of longitudinal studies, revealing that the relationships between age and cognitive performance are nuanced by the initial performance of the individuals. This aspect cannot be examined cross-sectionally; longitudinal data is essential for such insights. Similarly, baseline performance was found to influence the magnitude of change over time in longitudinal personality studies, too [47].

While dogs’ communication scores did not influence the common factor in the cross-sectional analysis, they appeared to play a more crucial role in longitudinal changes. Communication score was involved in two interactions: both with dogs’ baseline performance and with the time elapsed since the baseline measurement. The communication score included questions related to dogs’ sensitivity and attention to human visual and acoustic cues, as well as their communicative behavior towards humans (for exact questionnaire items, see Table S2 in SI2). Highly communicative dogs experienced a more negative impact on their performance as more time elapsed since the baseline measurement. However, the elapsed time did not affect the performance of dogs with lower communication scores. Cognitive performance might not solely depend on the amount of information acquired but also on the extent to which individuals rely on new information [64].

Dogs’ performance in object-choice tasks was found to be highly influenced by socially provided information, including unintentional or misleading cues [123–126]. However, dogs can be influenced to varying degrees in such tasks, which may be due to their different sensitivity and attention to human social cues [116, 126, 127]. It is probable that less communicative dogs are less willing to rely on humans instead of solving the problem on their own, which could positively affect their constant performance improvement through test experience. Dog training might be one factor that affects dogs’ sensitivity to human cues and dependence on humans in problem-solving tasks. Highly trained dogs were less prone to follow their owners’ misleading cues in a food choice task than untrained dogs [125]. Dogs’ training level and trainability were also found to be positively associated with cognitive performance in the cross-sectional analysis.

### Limitations

Several limitations of the current project should be acknowledged, particularly concerning the cognitive structure and the potential existence of a *g*-like factor in dogs.

Firstly, while our study demonstrates several improvements in terms of the diversity of cognitive tasks and their relevance to *g* in both humans and non-human animals, the analyses were still based on a relatively small set of tasks. Our research goal, and thus the protocol primarily aimed to investigate cognitive aging rather than to pinpoint the dog-equivalent of a general cognitive factor. Consequently, we selected tasks based on previous research indicating their age-related variations, which explains the limited task diversity. However, this constraint severely restricts the representation of various cognitive abilities and falls far short of human standards, where up to 20 sub-tests can serve as the basis for *g* extraction (see e.g. [128]). Notably, certain cognitive domains relevant to humans, such as reasoning by analogy, basic math, and language skills, remain unaddressed here, and it remains unclear if they can be convincingly measured in any non-human animals. The relatively low number of tasks also prevented us from identifying more than two domain-specific factors in contrast to the much more intricate human models [21, 22]. The low number of first-order constructs also hindered the exploration of alternative model structures like the nested factor model [129]. This scarcity of first-order factors might stem from insufficient overlap among the tasks regarding the cognitive domains they measured, with their common variance mostly accounted for by a domain–general *g* factor. Alternatively, the possibility of task impurity cannot be dismissed. Unlike human research, this line of research is still in its infancy in dogs, and our battery may include tasks unsuitable for assessing cognition. To mitigate these possibilities, we collected multiple variables for each task and extracted factors to represent the dogs’ aggregate performance. Despite our efforts, task impurities may still be present to some extent, obscuring the underlying structure.

Thirdly, alternative interpretations exist for the cognitive domains identified in our study. The individual problem-solving factor pertains to three tasks that require dogs to work independently from humans to obtain a food reward, suggesting that motivation level could serve as a non-cognitive source of variance for these tasks. However, the fact that the other two tasks (training for eye contact and clicker game) also involve obtaining food rewards, albeit through interaction with humans, suggests that a simple interest in food is unlikely to explain a substantial amount of variance in this factor. Regarding the two tasks forming the learning factor, both were conducted using a clicker-like device, suggesting that training experience could be a potential non-cognitive factor affecting their variance. To mitigate this potential bias, we employed a specific clicker device with a sound distinct from the traditional clicker device. Additionally, in the discrimination and reversal learning task, we found that the number of trials required to learn the initial discrimination (but not the reversal) significantly correlated with this factor, indicating that it reflects dogs’ learning ability, at least partially. However, we cannot entirely dismiss the possibility that some dogs scored higher in these tasks not solely due to their cognitive capacity but also because of their previous clicker training experience. Moreover, it is worth noting that the learning factor comprised only two tasks, so it cannot be considered stable [130]. Hence, further studies are necessary to determine whether associative learning indeed represents a distinct cognitive domain in dogs.

Finally, we cannot exclude the possibility that some of the positive correlations among cognitive tasks are attributable to similar relationships of the tasks with individual variables such as sex, breed, and health condition. Our investigation primarily focused on determining whether age affected the cognitive structure, as age appeared to be the most prominent candidate linked to multiple cognitive tasks in a similar manner. However, further studies are needed to account for additional demographic and keeping variables.

In summary, while the current study offers the most comprehensive insight into the cognitive structure of dogs to date, the common factor we identified serves as a preliminary approximation of a potential general cognitive factor rather than a definitive measure of *canine g*. It is imperative for future studies to examine a wider range of cognitive abilities, including additional candidate mechanisms such as response inhibition and processing speed, which are likely correlated with *g* [45] but were not addressed in this study. Moreover, alternative structural models should be investigated to determine whether our findings would hold in more complex models. Until then, definitive conclusions are not yet possible regarding the existence and precise structure of a *canine g*, and its analogy to the human *g* factor.

Further age-related effects may also emerge with the examination of a broader range of tasks or the establishment of more complex organizational structures. Additionally, the nature of shared age effects, whether they signify potential mediator effects or a shared (neurobiological) background, requires direct testing and clarification in future analyses.

Another set of limitations concerns the sample. While our sample size surpasses that of (some) previous studies (e.g., [44]), certain statistical analyses, such as the CFA, may still be somewhat underpowered. This lack of power is particularly evident in the longitudinal sample, where half of the eligible owners failed to return in each test session. The low response rate suggests that the subset of owners who participated in multiple longitudinal sessions may represent a smaller, more enthusiastic group of dog owners who may be interested in and interact more with their dogs compared to the average population. Furthermore, the high drop-out rate compelled us to analyze subjects with only two observations, despite the limitations inherent in two-wave studies: (1) all change is by default linear; (2) it is challenging to determine whether the change was consistent, delayed, or if it plateaued before changing again; (3) they may obscure genuine change and measurement error (e.g., test experience could enhance the performance of inexperienced subjects [131]). Consequently, some researchers argue that genuine longitudinal research should include a minimum of three repeated observations [132].

In addition to sample size, the constitution of the sample also presents some limitations. As mentioned earlier, the advanced age of the subjects could affect the assessment of long-term temporal consistency. However, perhaps the most significant limitation stemmed from excluding dogs with overt signs of age-related physical impairments. All subjects underwent a pre-screening process to ensure they were free from severe mobility issues, visual impairments, and hearing impairments. While this was necessary to ensure that their performance in the cognitive tasks was not significantly impacted by their physical condition, this approach limited our ability to detect severe cognitive decline in cases where deterioration in cognitive skills was not accompanied by observable health and sensory impairments.

### Conclusion

Despite these limitations, the current study fills important gaps in our knowledge regarding dog cognition and demonstrates new, intriguing parallels between human and dog aging, further strengthening the argument that dogs are an excellent model species in both research fields. The novel contribution of our research, compared to previous cognitive aging studies, lies in analyzing the age associations not only at the individual task level but also at higher levels of the hierarchy, and identifying distinct age-related influences.

Our findings also support the existence of a canine *g* factor, which opens the possibility of using dogs as a model species not only for cognitive aging but also to understand the nature, evolution, and underlying background factors of human intelligence. Dogs have already been recognized as valuable models regarding the genetic and neurobiological background of behavior and cognition [133, 134], and the environmental factors for humans and dogs can also be expected to largely overlap, offering higher translational value than many traditional model species except for primates. Moreover, some researchers have even suggested that dogs show higher similarity in cognitive structure to humans than primates, as during domestication, dogs were subjected to similar evolutionary pressures as humans, which have also affected how different social and cognitive abilities are related to each other [36].

However, it also needs to be acknowledged that inter-individual variation in cognitive performance could still have different sources in humans and dogs, not the least because selective breeding of dogs to different functions could have a substantial effect on the correlation pattern among the cognitive tasks. This, together with the above-listed limitations, advises caution when estimating the translational value of *canine g*, leaving future studies to determine how much the dog and human *g* factors are analogous. Nevertheless, this *g* factor candidate already demonstrated its utility in studying novel aspects of cognitive aging, which opens the door for future translational and veterinary advances in the field of cognitive aging.

## Supplementary Information

Below is the link to the electronic supplementary material.

Supplementary file1 (XLSX 120 KB)

Supplementary file2 (DOCX 54 KB)

Supplementary file3 (PDF 211 KB)

## Data Availability

All data are available in the supplementary materials.
