# Supplementary material for: Age-related effects on a hierarchical structure of canine cognition
Source: GeroScience. 2024 Mar 21;46(6):5843–74. doi: 10.1007/s11357-024-01123-1 (PMC11493892; doi:10.1007/s11357-024-01123-1)
Supplement: Supplementary file 2 — Supplementary file2 (DOCX 54 KB) [file 11357_2024_1123_MOESM2_ESM.docx]

**Supplementary Information 2**

**Title:** Age-related effects on a hierarchical structure of canine cognition

**Journal:** GeroScience

**Authors:** Zsófia Bognár, Borbála Turcsán*, Tamás Faragó, Dóra Szabó, Ivaylo Borislavov Iotchev, Enikő Kubinyi

**Affiliation of the corresponding author:** MTA-ELTE Lendület “Momentum” Companion Animal Research Group, Department of Ethology, Eötvös Loránd University, Budapest, Hungary

**email address of the corresponding author**: borbala.turcsan@gmail.com

**Table S1** Definition and inter-observer reliability (assessed by intraclass correlation (ICC)) of the variables coded in the Cognitive battery. A PCA was run on the coded variables for six tasks separately (all, except the Pointing task). Loadings of the variables, internal consistency and explained variance of the components, and task reliability assessments of the cognitive measures are also shown. The task reliability has been assessed using intraclass correlation (ICC) on N=72 dogs. Loadings > 0.5 are in bold. E: experimenter, O: owner

| **Task/Phase** | **Variable** | **Definition** | **ICC** | **p** | **Component 1** | **Component 2** |
| --- | --- | --- | --- | --- | --- | --- |
| COGNITIVE TASKS | | |  |  |  |  |
| **1. Pointing** | | |  |  |  |  |
| test trials | N of correct choices | Frequency of correct choices (pot with food) (out of 6) | 1.00 | <0.001 | *no PCA* |  |
| *Task reliability (ICC)* | | |  |  | *-0.081, p=0.626* |  |
| **2. Manipulative persistency** | | |  |  | *Persistency* |  |
| solvable | Time% look at the Kong | Duration of looking at the Kong | 0.99 | <0.001 | **0.914** |  |
| solvable | Time% touch the Kong | Duration of manipulating (touching, pawing, mouthing, nosing) the Kong | 0.96 | <0.001 | **0.907** |  |
| unsolvable | Time% look at the Kong | Duration of looking at the Kong | 0.99 | <0.001 | **0.916** |  |
| unsolvable | Time% touch the Kong | Duration of manipulating (touching, pawing, mouthing, nosing) the Kong | 0.99 | <0.001 | **0.915** |  |
| *Explained variance (%)* | | |  |  | *83.336* |  |
| *Cronbach's alpha* | | |  |  | *0.931* |  |
| *Task reliability (ICC)* | | |  |  | *0.840, p<0.001* |  |
| **3. Clicker game** | | |  |  | *Flexibility* | *One-trial learning* |
|  | N of repeated behaviors | Frequency of previously rewarded behaviors repeated (except looking at E) | 0.76 | <0.001 | 0.167 | **0.885** |
|  | N of novel behaviors | Frequency of new behaviors | 0.98 | <0.001 | **0.657** | 0.392 |
|  | Time% repeated behaviors | Duration of performing previously rewarded behaviors (except looking at E) | 0.97 | <0.001 | **0.754** | **0.566** |
|  | Time% passivity | Duration of staying still (sitting, lying, standing in one place) | 0.96 | <0.001 | **-0.798** | -0.158 |
|  | N of obedience tricks | Frequency of previously learned tricks performed, e.g., sit, lay down, giving paw, turning around, etc. | 0.89 | <0.001 | -0.049 | **0.930** |
| *Explained variance (%)* | | |  |  | *30.735* | *47.825* |
| *Cronbach’s alpha* | | |  |  | *0.592* | *0. 728* |
| *Task reliability (ICC)* | | |  |  | *0.594, p<0.001* | *0.764, p<0.001* |
| **4. Problem solving** | | |  |  | *Problem-solving success* | |
| opaque | Latency of finding the food, 1^st^ trial | From the moment the dog starts moving until the dog reaches into the box (or maximum: 30sec) | 0.92 | <0.001 | **-0.558** |  |
| opaque | Latency of finding the food, 2^nd^ trial | From the moment the dog starts moving until the dog reaches into the box (or maximum: 30sec) | 0.69 | <0.001 | **-0.782** |  |
| opaque | Latency of finding the food, 3^rd^ trial | From the moment the dog starts moving until the dog reaches into the box (or maximum: 30sec) | 0.86 | <0.001 | **-0.768** |  |
| transparent | Latency of finding the food, 4^th^ trial | From the moment the dog starts moving until the dog reaches into the box (or maximum: 30sec) | 0.99 | <0.001 | **-0.764** |  |
| transparent | Latency of finding the food, 5^th^ trial | From the moment the dog starts moving until the dog reaches into the box (or maximum: 30sec) | 0.93 | <0.001 | **-0.831** |  |
| transparent | Latency of finding the food, 6^th^ trial | From the moment the dog starts moving until the dog reaches into the box (or maximum: 30sec) | 0.88 | <0.001 | **-0.794** |  |
| transparent | Latency of finding the food, 7^th^ trial | From the moment the dog starts moving until the dog reaches into the box (or maximum: 30sec) | 0.96 | <0.001 | **-0.835** |  |
| transparent | Latency of finding the food, 8^th^ trial | From the moment the dog starts moving until the dog reaches into the box (or maximum: 30sec) | 0.82 | <0.001 | **-0.834** |  |
| transparent | Latency of finding the food, 9^th^ trial | From the moment the dog starts moving until the dog reaches into the box (or maximum: 30sec) | 0.92 | <0.001 | **-0.818** |  |
| transparent | Latency of finding the food, 10^th^ trial | From the moment the dog starts moving until the dog reaches into the box (or maximum: 30sec) | 0.84 | <0.001 | **-0.705** |  |
| all trials | N of correct first choices | Frequency of times the dog finds the food in the first choice (out of 10) | 0.70 | <0.001 | does not load >0.5 | |
| *Explained variance (%)* | | |  |  | *59.771* |  |
| *Cronbach's alpha* | | |  |  | *0.923* |  |
| *Task reliability (ICC)* | | |  |  | *0.523, p=0.001* |  |
| **5.** **Attention** | | |  |  | *Attention to Object* | |
| non-social | Latency to look away object | From the moment the frisbee starts moving until the dog looks away: 0: < 5 sec; 1: 5 - < 16 sec; 2: 16 - < 30 sec; 3: => 30 sec | 0.92 | <0.001 | **0.928** |  |
| non-social | Time% look at the object | 0: nearly 0%; 1:<50%; 2: =>50%; 3: nearly 100% of the time | 0.61 | 0.002 | **0.928** |  |
| social | Latency to look away human | From the moment the door opens until the dog looks away: 0: < 5 sec; 1: 5 - < 16 sec; 2: 16 - < 30 sec; 3: => 30 sec | 0.95 | <0.001 | does not load >0.5 | |
| *Explained variance (%)* | | |  |  | *86.050* |  |
| *Cronbach's alpha* | | |  |  | *0.817* |  |
| *Task reliability (ICC)* | | |  |  | *0.309, p=0.062* |  |
| **6. Training for eye contact** | | |  |  | *Associative learning* | |
| training | Mean eye contact latency | Mean latency of the first 15 eye contacts (from the moment the dog takes the sausage into its mouth until E clicks) (or maximum: 60 sec) | 1.00 | <0.001 | **-0.878** |  |
| training | Latency to learn | If the dog passed the training criteria, the sum of the first 15 eye contact's latency; if the dog did not pass the training criteria: 300 sec | 1.00 | <0.001 | **-0.936** |  |
| sustained | Sustained eye contact | Maximum duration of sustained eye contact: 0: did not pass first level; 1: 2 sec; 2: 5 sec; 3: 10 sec; 4: 20 sec; 5: 40 sec. Mean of the with and without distraction conditions | 0.64 | <0.001 | **0.848** |  |
| *Explained variance (%)* | | |  |  | *78.852* |  |
| *Cronbach's alpha* | | |  |  | *0.865* |  |
| *Task reliability (ICC)* | | |  |  | *0.870, p<0.001* |  |
| **7. Memory** | | |  |  | *Memory* |  |
|  | Latency of finding the food, 1^st^ trial | From the moment the dog starts moving until the dog's nose enters into the pot (or maximum: 30sec) | 0.99 | <0.001 | **-0.598** |  |
|  | Latency of finding the food, 2^nd^ trial | From the moment the dog starts moving until the dog's nose enters into the pot (or maximum: 30sec) | 0.90 | <0.001 | **-0.632** |  |
|  | Latency of finding the food, 3^rd^ trial | From the moment the dog starts moving until the dog's nose enters into the pot (or maximum: 30sec) | 0.88 | <0.001 | **-0.637** |  |
|  | Latency of finding the food, 4^th^ trial | From the moment the dog starts moving until the dog's nose enters into the pot (or maximum: 30sec) | 0.83 | <0.001 | **-0.686** |  |
|  | Latency of finding the food, 5^th^ trial | From the moment the dog starts moving until the dog's nose enters into the pot (or maximum: 30sec) | 0.95 | <0.001 | **-0.540** |  |
|  | N of correct first choices | Frequency of times the dog finds the food in the first choice (out of 5) | 1.00 | <0.001 | **0.823** |  |
| *Explained variance (%)* | | |  |  | *43.384* |  |
| *Cronbach's alpha* | | |  |  | *0.732* |  |
| *Task reliability (ICC)* | | |  |  | *0.679, p<0.001* |  |
| TESTS USED FOR VALIDATING THE COMMON FACTOR | | |  |  |  |  |
| **Exploration** | | |  |  | *Activity* |  |
| on leash | Time% activity | Moving the legs: 0: nearly 0%; 1: <50%; 2: =>50%; 3: nearly 100% of the time | 0.71 | <0.001 | **0.581** |  |
| free | Time% activity | Moving the legs: 0: nearly 0%; 1: <50%; 2: =>50%; 3: nearly 100% of the time | 0.87 | <0.001 | **0.891** |  |
| free | Time% proximity to O | being < 1 m to O: 0: nearly 0%; 1: <50%; 2: =>50%; 3: nearly 100% of the time | 0.75 | <0.001 | **-0.823** |  |
| free | N of objects visited | nose < 10 cm from object: 0: 0-1 objects; 1: 2-5 objects; 2: 6-10 objects; 3: 11-16 objects | 0.97 | <0.001 | **0.766** |  |
| *Explained variance (%)* | | |  |  | *43.104* |  |
| *Cronbach's alpha* | | |  |  | *0.757* |  |
| **Box rustle** | | |  |  | *Following* |  |
|  | Search box, 1^st^ | When O investigates the box, the dog 0: does not look; 1: looks but no approach; 2: approaches <1m but no touch; 3: touches the box | 0.88 | <0.001 | **0.752** |  |
|  | Search box, 2^nd^ | When O investigates the box, the dog 0: does not look; 1: looks but no approach; 2: approaches <1m but no touch; 3: touches the box | 0.78 | <0.001 | **0.718** |  |
|  | Search box, 3^rd^ | When O investigates the box, the dog 0: does not look; 1: looks but no approach; 2: approaches <1m but no touch; 3: touches the box | 0.75 | <0.001 | **0.765** |  |
|  | Time% following O | When O walks among the boxes, the dog follows O: 0: nearly 0%; 1: <50%; 2: =>50%; 3: nearly 100% of the time | 0.77 | <0.001 | **0.717** |  |
| *Explained variance (%)* | | |  |  | *54.499* |  |
| *Cronbach's alpha* | | |  |  | *0.651* |  |
| **Novel object recognition** | | |  |  | *Preference for novelty* | *Preference for familiar* |
| passive familiarization | N of approach any toys | Frequency of approaching any of the toys <20cm | 0.69 | <0.001 | **0.720** | 0.133 |
| passive familiarization | N of touch any toys | Frequency of touching any of the toys | 0.83 | <0.001 | **0.726** | 0.218 |
| passive familiarization | Time% proximity to any toys | Duration of being <20cm to any of the toys | 0.86 | <0.001 | 0.403 | **0.732** |
| passive familiarization | Time% touch any toys | Duration of touching any of the toys | 0.81 | <0.001 | 0.325 | **0.753** |
| test phase | N of approach familiar toy | Frequency of approaching the familiar toy <20cm | 0.65 | <0.001 | 0.145 | **0.609** |
| test phase | Time% proximity to familiar toy | Duration of being <20cm to the familiar toy | 0.93 | <0.001 | -0.230 | **0.947** |
| test phase | Time% proximity to new toy | Duration of being <20cm to the new toy | 0.93 | <0.001 | **0.868** | -0.153 |
| test phase | N of touch familiar toy | Frequency of touching the familiar toy | 0.53 | 0.008 | -0.052 | **0.750** |
| test phase | Time% touch familiar toy | Duration of touching the familiar toy | 0.85 | <0.001 | -0.237 | **0.900** |
| test phase | Time% touch new toy | Duration of touching the new toy | 0.92 | <0.001 | **0.871** | -0.149 |
| *Explained variance (%)* | | |  |  | *24.203* | *39.833* |
| *Cronbach’s alpha* | | |  |  | *0.848* | *0.886* |
| **Discrimination and reversal learning** | | |  |  |  |  |
| discrimination | Number of learning trials | The number of trials required to learn the initial association between the stimuli and reward until criteria; if the dog did not pass training criteria: 51 | 0.74 | <0.001 | *no PCA* |  |
| reversal | Number of reversal trials | The number of trials required to learn the reversed association between the stimuli and reward; if the dog did not pass training criteria: 51 | 0.86 | <0.001 | *no PCA* |  |

**Table S2** Questionnaire items and their answer options. A PCA was run on the Demographic factors, Owners' attitude, Cognition and communication, and Dogs' character parts of the questionnaire separately. Loadings of the items, internal consistency and explained variance of the components are also shown. Loadings > 0.4 are in bold

| **Variable** | | | **Answer type / options** | **Component 1** | **Component 2** | **Component 3** |
| --- | --- | --- | --- | --- | --- | --- |
| **Basic information about the dog and the owner** | | |  |  |  |  |
| dog's age | | | continuous |  |  |  |
| sex | | | male, female |  |  |  |
| reproductive status | | | intact, neutered |  |  |  |
| breed | | | purebred, mixed breed |  |  |  |
| weight | | | continuous |  |  |  |
| height at withers | | | continuous |  |  |  |
| body condition | | | underweight, normal, overweight |  |  |  |
| owner's age | | | 18-29 years, 30-39 years, 40-49 years, 50< years |  |  |  |
| previous experienced trauma (e.g. accident, surgery, lost for days, changed owners) | | | yes, no |  |  |  |
| **Keeping conditions** | | |  |  |  |  |
| Time spent playing with the owner per day | | | <1h, >1h |  |  |  |
| Off leash activity per day | | | <1h, 1-3h, >3h |  |  |  |
| Time spent alone per day | | | none, 1-2h, 3-8h, >8h |  |  |  |
| Other dogs in the household | | | none, one, more |  |  |  |
| **Demographic factors** | | |  | *Training level* | *Health issues* | *Family* |
| Number of tasks below the dog can reliably perform | | | maximum 3, 4, 5, 6, 7 or more | **0.849** | 0.044 | 0.045 |
| How many commands does your dog follow? | | | less than 10, 10 or more | **0.843** | 0.090 | 0.018 |
| Number of different training activities you are currently doing with your dog | | | 0, 1, 2 or more | **0.777** | -0.235 | -0.080 |
| Number of different training types the dog has received | | | 0, 1, 2 or more | **0.762** | 0.063 | 0.001 |
| Number of different leisure activities you are currently doing with your dog | | | 0, 1, 2 or more | **0.488** | -0.076 | 0.018 |
| How would you evaluate your experience with dogs? | | | minimal experience, moderate experience, professional | **0.414** | -0.111 | -0.048 |
| Does your dog get medication regularly? | | | yes, no | -0.022 | **-0.885** | 0.001 |
| Number of different health problems your dog has | | | none, 1, 2, 3 or more | -0.117 | **0.791** | 0.040 |
| Do you give your dog vitamins or supplements? (E.g. salmon oil, green-lipped mussel) | | | yes, no | 0.216 | **0.605** | -0.185 |
| Are there any children (under 12 years) living in the same household as your dog? | | | yes, no | -0.027 | -0.027 | **0.888** |
| How many people is your dog living together with? | | | 1, 2, 3, 4 or more | 0.072 | 0.016 | **0.879** |
| *Explained variance (%)* | | | | *21.00%* | *13.00%* | *11.00%* |
| *Cronbach's alpha* | | | | *0.723* | *0.620* | *0.533* |
| **Dog personality traits (DPQ)** | | | *45 items, 5-point Likert scale* |  |  |  |
| Fearfulness | | | 12 items, Cronbach's alpha: 0.793 |  |  |  |
| Aggression towards People | | | 6 items, Cronbach's alpha: 0.707 |  |  |  |
| Activity/Excitability | | | 12 items, Cronbach's alpha: 0.683 |  |  |  |
| Responsiveness to Training | | | 6 items, Cronbach's alpha: 0.646 |  |  |  |
| Aggression towards Animals | | | 9 items, Cronbach's alpha: 0.673 |  |  |  |
| **Owners' attitude towards dogs** *Do you agree with these statements? (5-point Likert scale)* | | | | *Emotional attitude* | *Doggy lifestyle* |  |
|  | My dog thinks like a child | | | **0.750** | -0.213 |  |
|  | I often pet and stroke my dog | | | **0.744** | 0.109 |  |
|  | My dog lets me know that he/she needs me | | | **0.725** | 0.028 |  |
|  | My dog makes me laugh and I play often with him/her | | | **0.655** | 0.254 |  |
|  | I always buy a gift for my dog too during the holidays | | | **0.631** | -0.018 |  |
|  | My dog is more important to me than any human | | | **0.520** | 0.028 |  |
|  | I try to get informed about professional dog training literature | | | -0.151 | **0.867** |  |
|  | I tend to talk a lot about dogs with my friends | | | 0.189 | **0.709** |  |
|  | Thanks to my dog I can get great possibilities to build new social connections | | | 0.133 | **0.681** |  |
|  | I bring my dog with me even it is not necessary | | | 0.153 | **0.555** |  |
| *Explained variance (%)* | | | | *29.00%* | *23.00%* |  |
| *Cronbach's alpha* | | | | *0,710* | *0.660* |  |
| **Cognition and communication** *How often does it occur that your dog… (4-point Likert scale)* | | | | *Communication* | *Signs of decline* | *Uncontrollability* |
|  | follows your gaze if you are looking at an object or into the distance | | | **0.741** | -0.016 | -0.074 |
|  | seeks eye contact with you | | | **0.721** | -0.038 | 0.018 |
|  | changes its behavior if you smile at it | | | **0.721** | -0.027 | -0.112 |
|  | spontaneously eavesdrops on human conversations and tries to decipher them? | | | **0.710** | 0.013 | 0.015 |
|  | pays attention to you, when you are talking to it | | | **0.691** | -0.219 | -0.158 |
|  | shows you something, or leads you to certain objects | | | **0.681** | 0.029 | 0.112 |
|  | copies your mood (e.g. your happiness, excitement is transferred to your dog)? | | | **0.652** | 0.079 | 0.017 |
|  | asks for your help if he/she cannot reach a goal alone E.g., when its ball rolls under the bed, the dog looks back and forth between the ball and you? | | | **0.646** | 0.155 | 0.164 |
|  | spontaneously tries to join in with what you are doing, and 'imitates' your actions? | | | **0.641** | 0.039 | 0.107 |
|  | follows pointing? E.g. when you point at an object your dog looks at it and/or moves towards it? | | | **0.588** | 0.011 | -0.125 |
|  | grins when greets, for example when meeting someone who the dog loves, the dog grins from ear to ear. | | | **0.582** | -0.024 | 0.014 |
|  | seeks out physical contact with you (cuddles up to you, leans against you) | | | **0.552** | -0.023 | 0.142 |
|  | invites you to play with him/her | | | **0.494** | -0.222 | 0.410 |
|  | tells you if he/she is hungry (barks, runs to the fridge, paw at or brings the food bowl) | | | **0.404** | 0.154 | 0.260 |
|  | gets lost in familiar places | | | -0.056 | **0.811** | -0.003 |
|  | knocks items accidentally or stumbles on the stairs | | | 0.027 | **0.795** | -0.108 |
|  | gets stuck somewhere (under the bed, in a corner) | | | -0.003 | **0.766** | 0.016 |
|  | has accidents in the house (e.g. urinating/defecating) | | | -0.048 | **0.670** | -0.010 |
|  | stares in one direction for "no reason" | | | 0.032 | **0.643** | -0.005 |
|  | trembles or shivers for no reason | | | 0.094 | **0.627** | -0.015 |
|  | does not respond to its name and seems not to realize it is being addressed | | | -0.247 | **0.601** | 0.100 |
|  | is sensitive to weather fronts | | | 0.179 | **0.598** | -0.110 |
|  | paces up and down or circles aimlessly (often in the same direction) | | | -0.038 | **0.537** | 0.266 |
|  | has problems eating or drinking | | | 0.034 | **0.536** | 0.010 |
|  | gets frightened by familiar people, does not recognize them | | | 0.031 | **0.508** | 0.127 |
|  | jumps up on people | | | -0.019 | -0.150 | **0.693** |
|  | is disobedient, does not follow your commands | | | -0.321 | 0.075 | **0.649** |
|  | gets too much absorbed in something (e.g. barking, digging, playing), and it is very hard to distract it? | | | 0.104 | 0.064 | **0.628** |
|  | actively tries to attract your attention (e.g. barking, nudging you with its nose etc.) | | | 0.276 | -0.041 | **0.595** |
|  | complains or throws tantrums if it gets frustrated? | | | 0.116 | 0.170 | **0.590** |
| *Explained variance (%)* | |  | | *20.00%* | *16.30%* | *8.30%* |
| *Cronbach's alpha* | |  | | *0.858* | *0.769* | *0.620* |
| **Dogs' character** *How typical is it of your dog to… (5-point Likert scale)* | | | | *Sociability/*  *trainability* | not used for analyses | |
|  | have recognizable feelings/emotions | | | **0.821** | 0.106 | 0.016 |
|  | follow you (from room to room) | | | **0.761** | 0.134 | 0.065 |
|  | be good at remembering things learned previously | | | **0.714** | -0.078 | -0.138 |
|  | be quick to learn new tasks | | | **0.690** | -0.229 | -0.083 |
|  | have good urine and faeces retention | | | **0.547** | -0.084 | -0.083 |
|  | exuberantly greet friends (human/dog) | | | **0.458** | -0.143 | 0.258 |
|  | sleep a lot during the day | | | 0.185 | **0.803** | 0.059 |
|  | have problems moving after getting up (general stiffness) | | | -0.072 | **0.719** | 0.073 |
|  | have a high need for movement | | | 0.242 | **-0.684** | 0.193 |
|  | be easily irritated | | | -0.085 | -0.031 | **0.751** |
|  | get frightened by objects or noises | | | 0.007 | 0.032 | **0.750** |
| *Explained variance (%)* | | | | *26.00%* | *16.00%* | *12.00%* |
| *Cronbach's alpha* | | | | *0,720* | *0.560* | *0.300* |

**Table S3** Relationship between the seven cognitive components and the age of the dogs analyzed with both the linear and quadratic regressions

| **Cognitive components** | **Regression** | **R^2^** | **F** | **df1** | **df2** | **p** |
| --- | --- | --- | --- | --- | --- | --- |
| Persistency | Linear | 0.017 | 2.213 | 1 | 126 | 0.139 |
|  | Quadratic | 0.019 | 1.215 | 2 | 125 | 0.300 |
| Flexibility | Linear | 0.038 | 5.018 | 1 | 126 | 0.027 |
|  | Quadratic | 0.046 | 3.028 | 2 | 125 | 0.052 |
| One-trial learning | Linear | 0.010 | 1.305 | 1 | 126 | 0.255 |
|  | Quadratic | 0.024 | 1.545 | 2 | 125 | 0.217 |
| Problem solving success | Linear | 0.025 | 3.185 | 1 | 126 | 0.077 |
|  | Quadratic | 0.037 | 2.415 | 2 | 125 | 0.093 |
| Associative learning | Linear | 0.110 | 15.632 | 1 | 127 | <0.001 |
|  | Quadratic | 0.111 | 7.834 | 2 | 126 | <0.001 |
| Memory | Linear | 0.135 | 19.826 | 1 | 127 | <0.001 |
|  | Quadratic | 0.151 | 11.238 | 2 | 126 | <0.001 |

**Table S4** Results of the unrotated exploratory factor analysis on the raw data and the (linear) age-residuals of the test data. Loadings > 0.3 are in bold

| **Cognitive component** | **Raw data** | | **Age-residuals** | |
| --- | --- | --- | --- | --- |
|  | *Factor 1* | *Factor 2* | *Factor 1* | *Factor 2* |
| Persistency | **0.636** | **-0.401** | **0.624** | **-0.330** |
| Flexibility | 0.134 | 0.236 | 0.057 | 0.189 |
| One-trial learning | **0.379** | **0.309** | **0.390** | **0.433** |
| Problem solving success | **0.704** | -0.118 | **0.724** | -0.099 |
| Associative learning | **0.452** | **0.376** | **0.362** | **0.334** |
| Memory | **0.569** | 0.033 | **0.511** | -0.045 |
| Initial Eigenvalue | 2.167 | 1.135 | 2.044 | 1.150 |
| Explained variance (%) | 26.498 | 7.806 | 24.336 | 7.599 |

**Table S5** Results of the unrotated exploratory factor analysis on components that compose the first factor, on the raw data and the (linear) age-residuals of the test data. Loadings > 0.3 are in bold

| **Cognitive component** | **Raw data** | **Age-residuals** |
| --- | --- | --- |
|  | *Factor 1* | *Factor 1* |
| Persistency | **0.607** | **0.615** |
| One-trial learning | **0.363** | **0.360** |
| Problem solving success | **0.715** | **0.728** |
| Associative learning | **0.509** | **0.399** |
| Memory | **0.558** | **0.510** |
| Eigenvalue | 2.140 | 2.039 |
| Explained variance (%) | 30.008 | 28.090 |
| Cronbach's alpha | 0.647 | 0.626 |
