# Supplementary material for: Age-related effects on a hierarchical structure of canine cognition
Source: GeroScience. 2024 Mar 21;46(6):5843–74. doi: 10.1007/s11357-024-01123-1 (PMC11493892; doi:10.1007/s11357-024-01123-1)
Supplement: Supplementary file 3 — Supplementary file3 (PDF 211 KB) [file 11357_2024_1123_MOESM3_ESM.pdf]

Supplementary Information 3

Title: Age-related effects on a hierarchical structure of canine cognition

Journal: GeroScience

Authors: Zsófia Bognár, Borbála Turcsán*, Tamás Faragó, Dóra Szabó, Ivaylo Borislavov Iotchev, Enikő Kubinyi

Affiliation of the corresponding author: MTA-ELTE Lendület "Momentum" Companion Animal Research Group, Department of Ethology, Eötvös Loránd University, Budapest, Hungary

email address of the corresponding author: borbala.turcsan@gmail.com

Fig. S1 Alternative model structures compared in confirmatory factor analysis. (A) Hierarchical g model; (B) No g model; (C) g-only model. In all models, the components were entered as observed variables (indicators) and are represented by rectangles. Latent factors are represented by ovals. Arrows from oval to rectangle indicate regression, and circles e1 to e7 represent error variance.

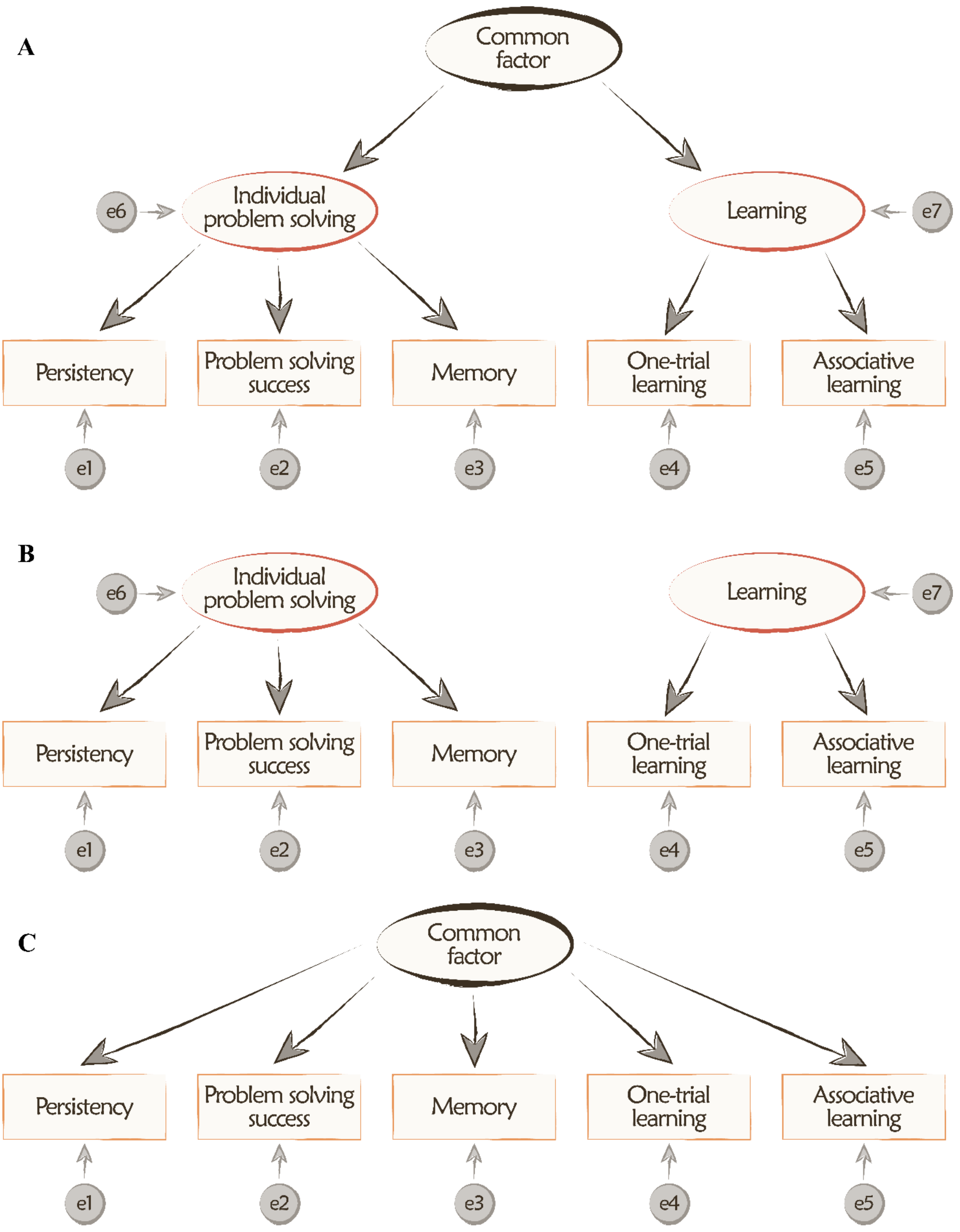
A
Common factor
Individual problem solving
Learning
e6
e7
Persistency
Problem solving success
Memory
One-trial learning
Associative learning
e1
e2
e3
e4
e5
B
Individual problem solving
Learning
e6
e7
Persistency
Problem solving success
Memory
One-trial learning
Associative learning
e1
e2
e3
e4
e5
C
Common factor
Persistency
Problem solving success
Memory
One-trial learning
Associative learning
e1
e2
e3
e4
e5
